# Defining ICR-Mo, an intrinsic colistin resistance determinant from *Moraxella osloensis*

**DOI:** 10.1371/journal.pgen.1007389

**Published:** 2018-05-14

**Authors:** Wenhui Wei, Swaminath Srinivas, Jingxia Lin, Zichen Tang, Shihua Wang, Saif Ullah, Vishnu Goutham Kota, Youjun Feng

**Affiliations:** 1 Department of Medical Microbiology & Parasitology, Zhejiang University School of Medicine, Hangzhou, Zhejiang, China; 2 School of Life Sciences, Fujian Agriculture and Forestry University, Fuzhou, China; 3 Department of Biochemistry, University of Illinois at Urbana-Champaign, Illinois, United States of America; 4 College of Animal Sciences, Zhejiang University, Hangzhou, Zhejiang, China; Uppsala University, SWEDEN

## Abstract

Polymyxin is the last line of defense against severe infections caused by carbapenem-resistant gram-negative pathogens. The emergence of transferable MCR-1/2 polymyxin resistance greatly challenges the renewed interest in colistin (polymyxin E) for clinical treatments. Recent studies have suggested that *Moraxella* species are a putative reservoir for MCR-1/2 genetic determinants. Here, we report the functional definition of ICR-Mo from *M*. *osloensis*, a chromosomally encoded determinant of colistin resistance, in close relation to current MCR-1/2 family. ICR-Mo transmembrane protein was prepared and purified to homogeneity. Taken along with an *in vitro* enzymatic detection, MALDI-TOF mass spectrometry of bacterial lipid A pools determined that the ICR-Mo enzyme might exploit a possible “ping-pong” mechanism to accept the phosphoethanolamine (PEA) moiety from its donor phosphatidylethanolamine (PE) and then transfer it to the 1(or 4’)-phosphate position of lipid A via an ICR-Mo-bound PEA adduct. Structural decoration of LPS-lipid A by ICR-Mo renders the recipient strain of *E*. *coli* resistant to polymyxin. Domain swapping assays indicate that the two domains of ICR-Mo cannot be functionally-exchanged with its counterparts in MCR-1/2 and EptA, validating its phylogenetic position in a distinct set of MCR-like genes. Structure-guided functional mapping of ICR-Mo reveals a PE lipid substrate recognizing cavity having a role in enzymatic catalysis and the resultant conference of antibiotic resistance. Expression of *icr-Mo* in *E*. *coli* significantly prevents the formation of reactive oxygen species (ROS) induced by colistin. Taken together, our results define a member of a group of intrinsic colistin resistance genes phylogenetically close to the MCR-1/2 family, highlighting the evolution of transferable colistin resistance.

## Author summary

This work represents the first functional definition of ICR-Mo, a member of a family of MCR-1/2 like genes from *Moraxella*. As inferred by phylogeny, this is the closest family of chromosomally encoded determinants of colistin resistance. We show evidence that ICR-Mo exploits a possible “ping-pong” mechanism to catalyze the transfer of PEA from its donor PE to the 1(or 4’)-phosphate position of lipid A via an adduct of ICR-Mo-bound PEA. Structure-guided functional studies reveal that ICR-Mo possesses a PE lipid substrate recognizing cavity having a role in enzymatic catalysis and the resulting bestowal of antibiotic resistance against colistin. The expression of *icr-Mo* in *E*. *coli* prevents the generation of ROS in response to treatment with colistin. The fact that the two domains of ICR-Mo cannot be functionally exchanged with their counterparts in MCR-1/2 validates its standing as a distinct phylogenetic entity. In summary, the results define a closer chromosomal relative of MCR-1/2 variants than EptA and highlight its role in the evolution of transferable colistin resistance.

## Introduction

Prevalent antibiotic resistance is posing a threat to providing safe and effective health care worldwide. Antimicrobial resistance (AMR) is associated with 700,000 deaths each year [1, 2]. It is estimated by O’Neill and his team that AMR would claim as many as 10,000,000 deaths per year globally by 2050 [3]. Despite this prediction being exaggerated and unreliable [4], we acknowledge that AMR has been posing an ever-growing burden on clinical therapies and public health, thereby highlighting the urgent need for a nationwide response to alleviate this burden [4, 5]. Polymyxins, a class of cationic cyclic polypeptide antibiotics, act as a “last-resort” option against infections by carbapenem-resistant gram-negative pathogens [6–9]. However, the emergence and global spread of plasmid-borne mobilized colistin resistance determinants (*mcr-1*) has greatly threatened the renewed interest of colistin (polymyxin E) in clinical therapies [10]. To the best of our knowledge, *mcr-1*-harboring Enterobacteriaceae have been detected in no less than 40 countries worldwide, spanning 5 of 7 continents [11], including the United States of America [12, 13].

MCR-1 is annotated as a transmembrane PEA transferase, which belongs to the “YhjW/YjdB/YijP” alkaline phosphatase super-family [10, 14]. MCR-1 catalyzes the transfer of phosphoethanolamine (PEA) from phosphatidylethanolamine (PE) to the 1(or 4’)-phosphate position of lipid A glucosamine (GlcN) moieties (**S1 Fig**) [15–17]. In general, the addition of PEA to lipid A is believed to reduce the net negative charge of the bacterial outer-membrane [6, 18, 19], consequently leading to a phenotypic resistance to polymyxin, a final line of defense against severe infections by pathogens with multi-drug resistance (MDR) [7, 8]. In comparison to the paradigm PEA transferase, the *Neisseria* EptA protein [16, 20], MCR-1 seems to render the recipient *E*. *coli* strains more resistant to colistin [14], implying that it is a more efficient enzyme. In contrast to the more prevalent MCR-1, its cousin MCR-2 with almost 80% amino acid identity, seems to be rare in that it is only detected in Belgium in 2016 [21]. In particular, it seems likely that both MCR-1 and MCR-2 have an evolutionarily-conserved catalytic motif having essential roles in enzymatic activities and resultant antibiotic resistance [14, 22].

Along with other transferable determinants of colistin resistance (MCR-3 [23, 24], MCR-4 [25] and MCR-5 [26]), a growing body of MCR-1 variants with point substitutions have been reported, such as MCR-1.2 [Q3L] [27], MCR-1.3 [I38V] [28] and MCR-1.6 [R536H] [29]. More recently, *mcr-1* and *mcr-2* variant genes have been discovered on chromosomes of *Moraxella* species [30, 31]. In brief, MCR-1.10 with 98.7% amino acid identity to MCR-1 is present in *M*. *porci* MSG13-C03 [30], MCR-2.2 with 99% amino acid identity (only 8 amino acid substitutions of 538 residues) to MCR-2 is encoded on the chromosome of *M*. *pluranimarium* [31], and MCR-2.3 with 87.9% amino acid identity to MCR-2 is harbored in a *M*. *pluranimalium*-like isolate, MSG47-C17. Moreover, it seems that MCR-like enzymes with about 60% amino acid identity are prevalent in *Moraxella* species [32], suggesting a potential reservoir of *mcr*-like genes. However, this requires further experimental evidence.

In this study, we have integrated multiple lines of approaches to study the structure and mechanism for one such *mcr*-like gene, AXE82_07515 in *M*. *osloensis* (hereafter designated as *icr-Mo* [of note: Mo denotes *Moraxella osloensis*]). This will allow it to serve as a representative member of this family for future studies. Consistent with genetic speculations raised by other groups [30–32], our results formulate a functional definition of *icr-Mo* and provide an evolutionary relationship between itself, *mcr-1/2* variants and non-*mcr* genes that confer colistin resistance. By probing the biochemical and physiological relevance, our findings highlight the importance of the *Moraxella* family in understanding the growing body of *mcr-1/2*-like genetic determinants conferring colistin resistance.

## Results

### Evolution of *mcr*-like genes in *Moraxella*

**Fig 1:**
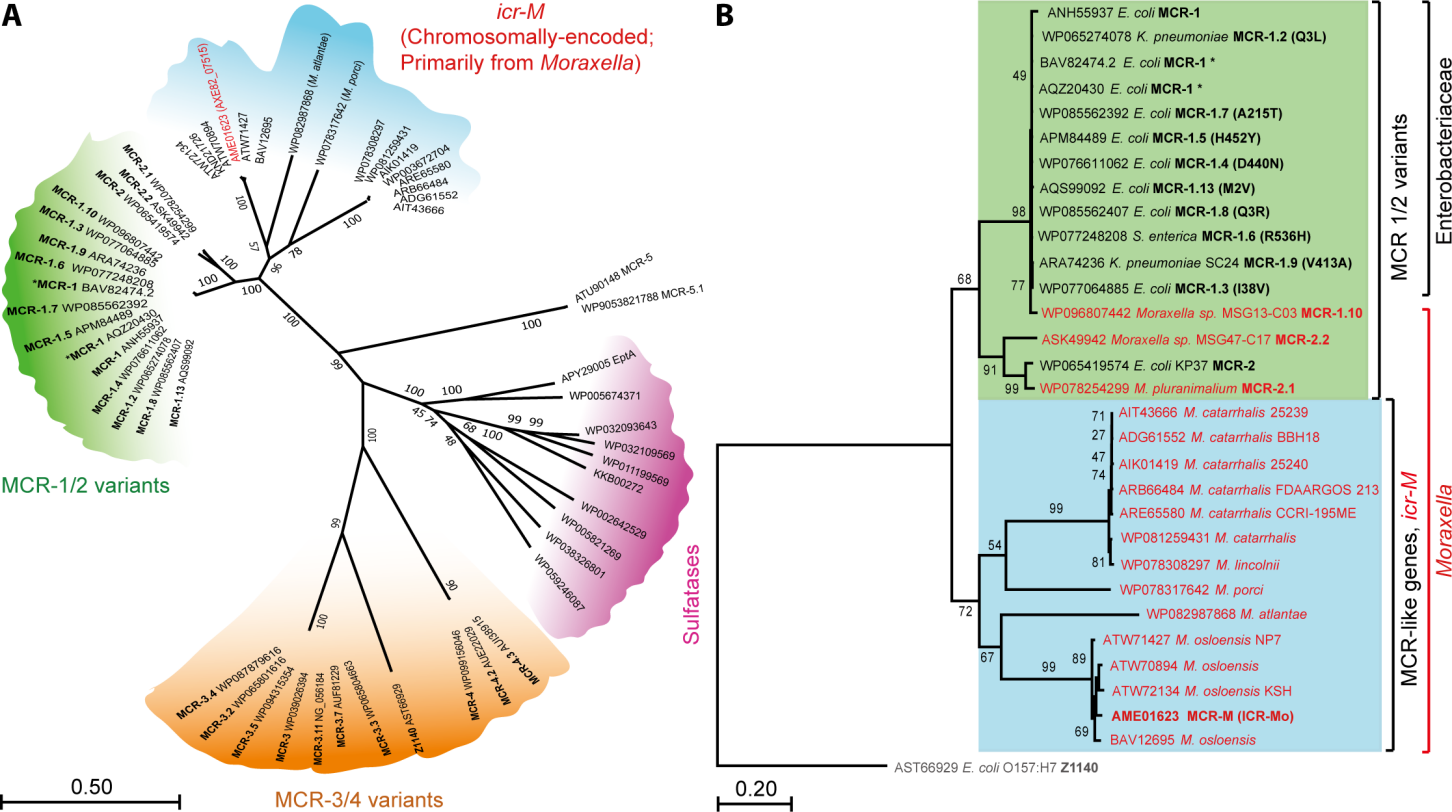
Phylogeny of *icr-Mo* (AXE82_07515). **A.** The unrooted nucleotide-based radial phylogram of *icr-Mo*. Nucleotide sequences of putative sulfatases homologous to MCR-1 are included in the analysis as a reference for evolutionary distance. Four distinct subclades included i) MCR-1/2 variants (in green); ii) *icr-Mo* (a chromosomally-encoded homologue of *mcr*-1/2 variants), which are mostly from (but not limited to) the *Moraxella* species; iii) Sulfatases homologous to MCR-1 (in pink) and iv) MCR-3/4 variants (in Orange). In particular, a chromosomally-encoded colistin resistance determinant, *Neisseria eptA* falls under the evolutionarily-distant Sulfatase cluster. Unlike ICR-Mo (AXE82_07515), a functional PE transferase (in red), Z1140 is an experimentally-verified non-functional PE transferase (**S2 Fig**) and acts as an internal reference in this phylogeny. **B.** Phylogenetic tree of *icr-Mo* (AXE82_07515) and its close homologs. Two distinct subclades are clustered, including i) MCR-1/2 variants (in the green background) and ii) Chromosomal MCR-like variants (in blue background, with genes from *Moraxella* species highlighted in red). The tree has been rooted with Z1140, an experimentally-verified non-functional PE transferase (**S2 Fig**). An asterisk has been used to indicate MCR-1 sequences with a silent DNA point mutation. The nucleotide sequence-based phylogeny of ICR-Mo homologs was inferred using the maximum likelihood method and a GTR nucleotide substitution model. The percentages of replicate trees in which the associated taxa are clustered in the bootstrap test (1000 replicates) is shown next to the branches. A discrete gamma distribution was used to model evolutionary rate differences among sites with some evolutionarily invariable sites. Accession numbers corresponding to the nucleotide sequence used have been indicated in the figure.

The gene *mcr-1*, is a prevalent determinant of plasmid-borne mobilized colistin resistance with a global distribution. In addition to a closely-related but rare *mcr-1* homolog, *mcr-2* sharing 81% protein identity [21], a number of genetic variants of *mcr-1* (like *mcr-1*.*2* [27]) with point substitutions have also been elucidated. Earlier this year, Kieffer and coworkers [32] showed genetic evidence that *Moraxella* species might be a potential reservoir for *mcr*-like genes. This prediction is further validated by additional discovery of *mcr-1*.*10* (98.7% amino acid identity) in *M*. *porci* MSG13-C03 [30] and *mcr-2*.*1* (99% amino acid identity) in *M*. *pluranimarium* [31]. In addition to their similarity, certain members of these chromosomally encoded *mcr-1/2*-like genes also neighbor almost identical mobilizable elements like the insertion sequence IS*Apl1*, associated with *mcr-1/2* dissemination [31, 32]. To probe the evolutionary relationships between the *icr-Mo* in *Moraxella* and other extant *mcr* genes, we performed phylogenetic analyses (**Fig 1**), using their coding nucleotide sequences. As anticipated, MCR-1 and its variants (namely MCR-1.2, MCR-1.3, … MCR-1.9 and MCR-1.10) cluster together to form a tight group (**Fig 1A** and **1B**). In fact, we have indicated 2 additional variants of MCR-1 with single synonymous point mutations. MCR-2 and its two variants (MCR-2.1 and MCR-2.2) also form a distinct group (**Fig 1A** and **1B**) while still being closely-clustered within a single subclade, comprising MCR-1/2 variants (**Fig 1A** and **1B**). Surprisingly, *Neisseria* EptA, a chromosomal colistin resistance determinant (31.5% amino acid identity to ICR-Mo, **S3 Fig**) clusters with other putative sulfatases, a large family of enzymes encoding broad functionality that includes PEA transferases. The sulfatase sub-clade along with a cluster comprising MCR-3 and MCR-4 variants are only distantly-related to the MCR-1/2 and MCR-like genes. In fact, Z1140 (539 aa) of *E*. *coli* O157:H7, a putative PEA transferase lacking detectable ability to confer phenotypic resistance to polymyxin (**S2 Fig**) is found within the MCR-3/4 subclade leading us to categorize them as non MCR-like genes (**Fig 1A**). Given that the identity between ICR-Mo and EptA is only 31.5%, it seems likely that *mcr-1/2* and *icr-Mo* genes have diverged from sulfatases much earlier than the recent colistin usage in agriculture and clinical medicine. A much closer ancestor might be found in bacteria that naturally produce polymyxins as secondary metabolites.

In contrast, a subset of MCR-like members exclusively from the *Moraxella* species (*porci*, *lincolnii and catarrhalis* subspecies, in **Fig 1**) with about 60% amino acid identities (**S3 Fig**) are clustered into a neighboring clade that includes ICR-Mo (**Fig 1A** and **1B**). This also includes the recently crystallized ICR-Mc (Accession No: AIT43666) from *Moraxella catarrhalis* which shares 55.2% amino acid identity with ICR-Mo [33]. We are inclined to believe that plasmid-borne MCR-1/2 variants and the intrinsic ICR-Mo might share a common ancestor. Intriguingly, certain species of *Moraxella* contain chromosomally encoded colistin resistance determinants that are grouped with members of MCR-1/2 family (**Fig 1B**). Therefore, a systematic evaluation of biochemical and physiological properties of ICR-Mo, is necessary.

### Characterization of ICR-Mo

Prediction with TMHMM server v2.0 (http://www.cbs.dtu.dk/services/TMHMM) suggests that ICR-Mo is an integral membrane protein possessing five N-terminal helices (**S3A Fig**), which is almost identical to those of MCR-1 [14] and MCR-2 [22]. As recently described for MCR-1/2 [14, 22], the transmembrane protein ICR-Mo was overexpressed and purified to homogeneity with nickel affinity column in the presence of 1% detergent dodecyl-β-D-maltoside (DDM). Following visualization by 12% SDS-PAGE (**S4A** and **S8A Figs**), identity of the recombinant ICR-Mo protein was determined with MALDI-TOF MS (Matrix-Assisted Laser Desorption/Ionization Time of Flight Mass Spectrometry) with a 84.67% fragment sequence (**S10C Fig**). The structure of full-length EptA (PDB: 5FGN) was used as a template [34] for the structural modeling of ICR-Mo via Swiss-Model (https://swissmodel.expasy.org/interactive) [35]. Despite possessing only 31.5% amino acid identity to EptA (**S2 Fig**), the modeled structure of ICR-Mo shows an appreciable coverage of 93% (24–558), indicating a satisfied prediction (**S4 Fig**). ICR-Mo also displays a similar topology to EptA (**S4B Fig**) [34]. The TM domain spanning the inner-membrane involves five α-helices (**S4B Fig**), and the catalytic domain has a hydrolase-fold (**S4C Fig**) comprising 10 α-helices and 7 β-sheets (**S4C Fig**). The two domains are linked by four short periplasm loops (PH2, PH2’, PH3 and PH4), a bridge helix (BH) and a long-coiled loop (**S4B** and **S4C Fig**).

### Enzymology of ICR-Mo

**Fig 2:**
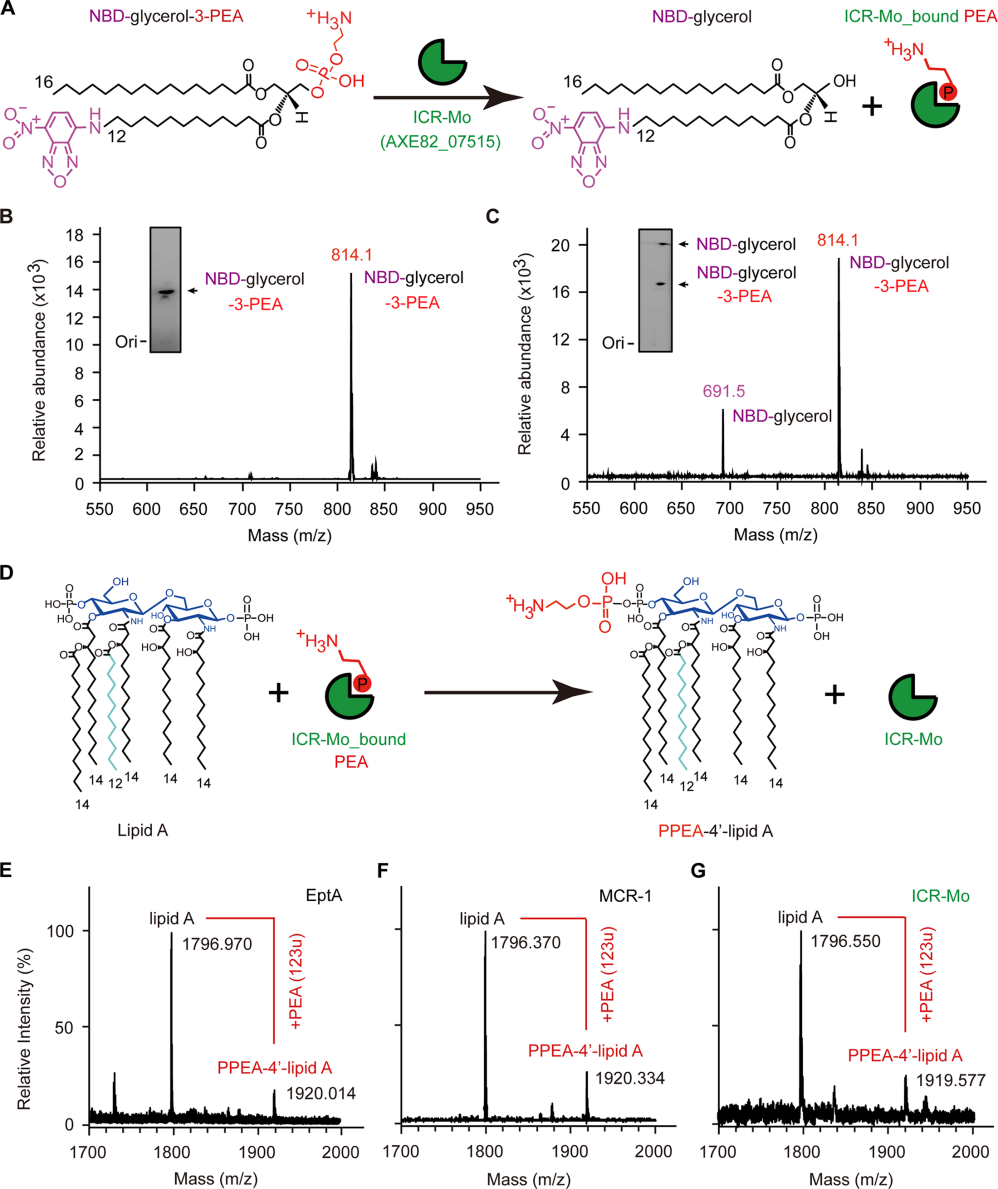
Chemical mechanism for PEA modification of lipid A by EptA/MCR-1/ICR-Mo. **A.** Scheme for cleavage reaction of an alternative substrate NBD-glycerol-3-PEA by ICR-Mo (AXE82_07515) into NBD-glycerol and an adduct of ICR-Mo-bound PEA. PEA refers to phosphoethanolamine. NBD was highlighted in magenta, whereas PEA was indicated in red. LC/MS identification of the mixture of the ICR-Mo-mediated hydrolysis reaction (**B**) and the NBD-glycerol-3-PEA substrate (**C**). The inside gels denote the TLC-based visualization of the NBD-glycerol-3-PEA substrate (in **Panel B**) and the ICR-Mo-mediated hydrolytic product, NBD-glycerol (in **Panel C**). NBD-glycerol-3-PEA appears at m/z of 814.1, whereas the resultant product NBD-glycerol occurs at m/z of 691.5. **C.** Thin layer chromatography (TLC) detection for the conversion of NBD-glycerol-PEA lipid substrate by the ICR-Mo (AXE82_07515) into NBD-glycerol. **D.** Transfer of PEA from ICR-Mo-bound PEA to lipid A, generating the PPEA-lipid A product. Position of PPEA depicted is only suggestive. MALDI-TOF-MS evidence for the structural alteration of the lipid A moieties of lipopolysaccharide (LPS) in *E*. *coli* expressing EptA (**E**), MCR-1 (**F**) and ICR-Mo (AXE82_07515) (**G**). The peak of the bis-phosphorylated hexa-acylated lipid A varies at m/z of 1796.063 ~ 1797.426, whereas resultant derivative with PEA modification (PPEA-1(or 4’)-lipid A) exhibits at m/z varying from 1919.409 to 1920.087.

As described by Anandan *et al*. [34] for EptA activity, we established an *in vitro* system for ICR-Mo enzymatic reaction. In this reaction system, the PEA donor substrate used as a fluorescent label, 1-acyl-2-{12-[(7-nitro-2-1,3-benzoxadiazol-4-yl) amino] dodecanoyl}-sn-glycerol-3-phosphoethanolamine (abbreviated as NBD-glycerol-3-PEA, **Fig 2A**). It is hypothesized that ICR-Mo removes the PEA moiety from the alternative substrate NBD-glycerol-3-PEA, and gives rise to a NBD-glycerol product (**Fig 2A**). Given that an adduct of PEA-Thr280-EptA has been observed by Vrielink and coworkers [20], we anticipate that an intermediate of ICR-Mo-bound PEA (i.e., PEA-Thr287-ICR-Mo) is produced in the hydrolytic reaction (**Fig 2A**). Thereafter, we utilized thin layer chromatography (TLC) to separate the mixture of the ICR-Mo reaction (**Fig 2B** and **2C** and **S5 Fig**). As expected, the product NBD-glycerol is released in the presence of ICR-Mo, which is consistent with observations of EptA and MCR-1/2 (**S5A** and **S5B Fig**) [36, 37]. Under blue light (455–485 nm), TLC-based assay illustrates that the NBD-glycerol product (**Fig 2C**) migrates faster than the substrate NBD-glycerol-3-PEA does (**S5 Fig**, **Fig 2B** and **2C**). Subsequently, liquid chromatography mass spectrometry (LC/MS) confirms the identities of NBD-glycerol-3-PEA (**Fig 2B**) and its resultant product, NBD-glycerol (**Fig 2C**). This demonstrates clearly that ICR-Mo displays an enzymatic activity of removing the PEA moiety from the lipid substrate *in vitro*, which is similar to the well-studied EptA [34], as well as MCR-1/2 (**S5 Fig**).

To further the *in vivo* transfer of PEA from PE lipids (**S1A Fig** and **Fig 2D–2G**), we engineered *E*. *coli* MG1655 strains to carry arabinose inducible plasmid pBAD24-borne *icr-Mo* (and *eptA/mcr-1*). We prepared and purified the LPS-lipid A from an array of *E*. *coli* strains. Unlike the negative control MG1655 exhibiting a single peak (m/z of 1796.370–1797.843) for lipid A alone (**Fig 2E–2G**), MALDI-TOF mass spectrometry unveiled an additional peak associated with PPEA-1(or 4’)-lipid A, the modified form of lipid A and this peak appeared in the *E*. *coli* strain harboring *mcr*-like genes. We believe that lipid A accepts PEA moiety from the intermediate of ICR-Mo-bound PEA, giving PPEA-4’-lipid A (**Fig 2D**). In fact, the molecular mass of PPEA-lipid A in different strains exhibits slight variation, namely m/z 1920.014 for EptA (**Fig 2E**), m/z 1920.334 for MCR-1 (**Fig 2F**), and m/z 1919.577 for ICR-Mo (**Fig 2G**). The *in vitro* and *in vivo* evidence leads us to believe that ICR-Mo catalysis proceeds by PEA transfer from the donor PE lipid substrate to the receiver lipid A, giving the two final products, PPEA-4’-lipid A and diacyl glycerol (**S1A Fig**). Taking into account the similarity between the adduct of Thr280-PEA in EptA [20, 34] and its counterparts in the reactions involving alkaline phosphatase-type phosphate transferase [38], it can be hypothesized that i) the ICR-Mo-bound PEA intermediate is released from the PE lipid molecule in the first-half reaction of ICR-Mo catalysis (**S1B Fig**); ii) In the second half-reaction, PEA is transferred from the ICR-Mo-bound PEA adduct to the 1(4’)-phosphate position of lipid A GlcN moieties, generating PPEA-4’- lipid A (**S1C Fig**). It seems likely that ICR-Mo exploits a possible “ping-pong” mechanism for enzymatic catalysis, similar to those proposed for EptA [34] and MCR-1/2 [36, 37, 39].

### A cavity in ICR-Mo for the entry of PE substrate

**Fig 3:**
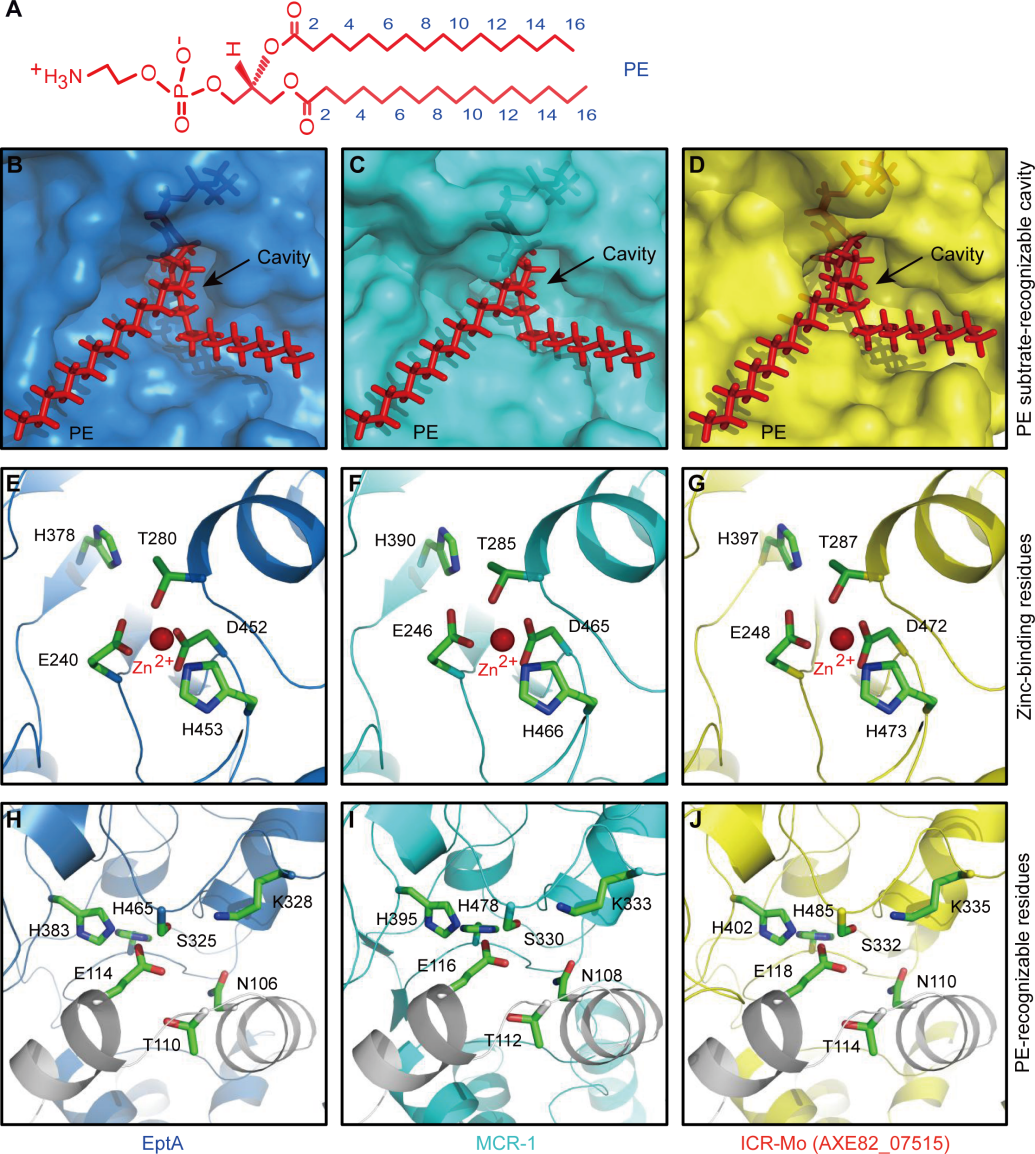
Paralleled PE-recognizing cavities amongst ICR-Mo, EptA and MCR-1. **A.** Chemical structure of the PE lipid substrate molecule. **B.** EptA has a cavity for the entry of the PE lipid substrate. **C.** A PE-recognizable cavity is present in MCR-1 enzyme. **D.** A conservative PE-binding cavity is also shared by ICR-Mo (AXE82_07515). A Zn^2+^-bound five-residues forming motif is conserved in PE lipid substrate-interactive cavities of three enzymes EptA (**E**), MCR-1 (**F**) and ICR-Mo (**G**). Comparative analyses of the seven conserved PE-recognizable residues from EptA (**H**), MCR-1 (**I**) and ICR-Mo (**J**). The enlarged surface structures of PE-bound cavities are consistently generated through molecular docking together with structural modelling. PE molecules are illustrated with red sticks, and cavity is highlighted with an arrow. The photographs are generated using PyMol. The conserved residues are labelled, and also listed in **S2 Fig**. Designations: PE: phosphatidylethanolamine.

On the basis of the newly-determined complex structure of EptA with the detergent DDM, an analogue of its physiological PE substrate [34], we applied molecular docking to reanalyze the binding to PE molecule (**Fig 3A**). As a result, it allows us to propose a 12 residue-constituting cavity for PE entry/binding (**Fig 3**). Superposition of the modeled structures of MCR-1 (ICR-Mo) to EptA also gives a similar PE-recognizable cavity (**Fig 3C** and **3D**). Amongst them, five amino acids (E248, T287, H397, D472 and H473) are centering with the zinc ion (**Fig 3E–3G** and **S7A** and **S7B Fig**), and remaining 7 residues (N110, T114, E118, S332, K335, H402 and H485) are anticipated to be involved in the recognition of its physiological lipid PE substrate (**Fig 3H–3J** and **S7C** and **S7D Fig**). Similar observations have been made earlier for K333, H395 and H478 in MCR-1 [40–44]. This raises a possibility that it is an evolutionarily-conserved functional cavity shared amongst the family of MCR-like enzymes (**Fig 3**). However, this hypothesis needs further experimental validation.

**Fig 4:**
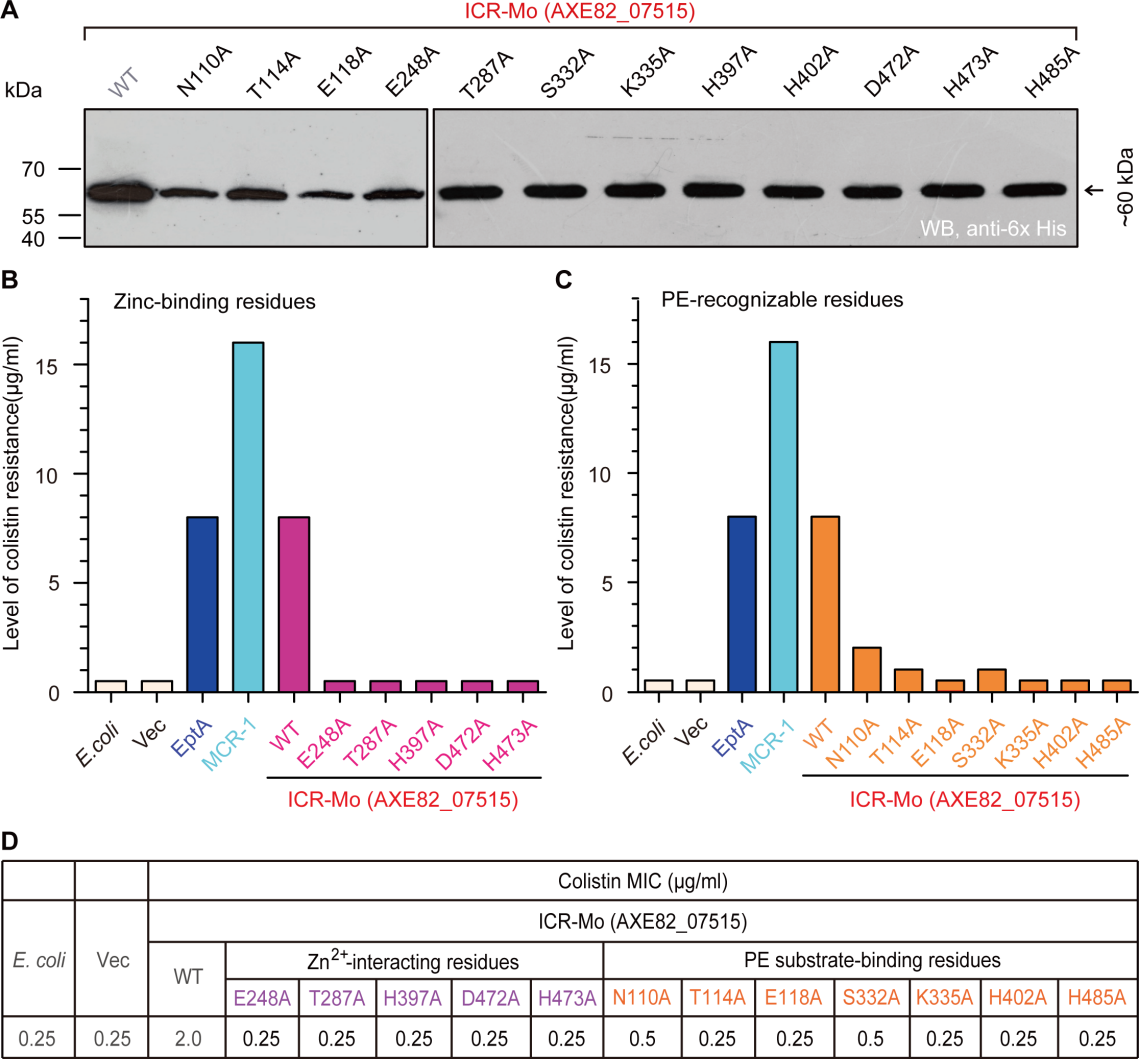
Functional analyses for the Zinc, PE-interacting motifs of substrate-bound cavities amongst EptA, MCR-1 and ICR-Mo (AXE82_07515). **A.** Western blot analyses of expression of ICR-Mo and its 12 point-mutants in *E*. *coli*. **B.** Structure-guided site-directed mutagenesis analyses for the Zn^2+^-binding motif of ICR-Mo using the LBA plate-based assays of colistin resistance. The five residues (E248, T287, H397, D472 and H473) are required for the binding of ICR-Mo to the zinc ion. **C.** Functional mapping of the PE-interactive residues of ICR-Mo in the context of colistin resistance. **D.** The measurement of colistin MIC of the *E*. *coli* strains carrying the wild-type *icr-Mo* (and/or its point-mutants). Level of colistin resistance was tested the LBA plates. A representative result is given from no less than three independent trials. In terms of level of colistin resistance, AXE82_07515 is almost identical to EptA, but only half of that associated with MCR-1. Designations: Vec, an empty vector pBAD24; WT, the wild-type of ICR-Mo (AXE82_07515).

We thereafter conducted structure-guided site-directed mutagenesis, generating 12 point-mutants of ICR-Mo (**S2 Fig** and **Figs 3E–3J** and **4A**). Prior to functional assays *in vivo*, western blotting demonstrates that all the mutated versions of ICR-Mo express well in *E*. *coli* (**Fig 4A**). Assays of colistin resistance elucidate that (i) ICR-Mo permits the recipient *E*. *coli* to grow on LBA plates with up to 8 μg/ml colistin [which is equivalent to that of EptA (**S6 Fig**), but significantly lower than MCR-1 does (16 μg/ml)] (**Fig 4** and **S6 Fig**); (ii) all the 5 point-mutants of ICR-Mo with defects in zinc-binding residues fail to support the growth of the recipient *E*. *coli* strains under the condition with up to 0.5 μg/ml colistin (**Fig 4B**); (iii) Among the 7 ICR-Mo mutants with the alanine substitution in putative PE substrate-interacting residues, 3 of them seem to retain partial activity (namely N110A [2 μg/ml], T114A [1 μg/ml] and S332A [1 μg/ml], **Fig 4C**) and the remaining 4 mutants are nonfunctional [0.5 μg/ml] (**Fig 4C**). These results are in agreement with the observations in the colistin MIC measurements (**Fig 4D**).

### Biochemical analyses of PE-interactive cavity in ICR-Mo

To gain a mechanistic glimpse of the PE substrate-interactive cavity, we purified the 12 point-mutants of ICR-Mo protein to homogeneity (**S8A Fig**). First, catalytic activities of the ICR-Mo mutants were examined using the *in vitro* enzymatic reaction with the fluorescence-labelled substrate, NDB-glycerol-3-PEA (**S5A** and **S8B Figs**) [34]. As anticipated, our TLC results elucidate that i) Two mutants of ICR-Mo [N110A and S332A] retain partial activities of hydrolyzing of NBD-glycerol-3-PEA into NBD-glycerol (**S8B Fig**); and ii) The other 10 mutated versions of ICR-Mo have no detectable enzymatic activity [*i*.*e*., an alternative substrate NDB-glycerol-3-PEA is not converted into NDB-glycerol, almost identical to the negative controls] (**S8B Fig**).

**Fig 5:**
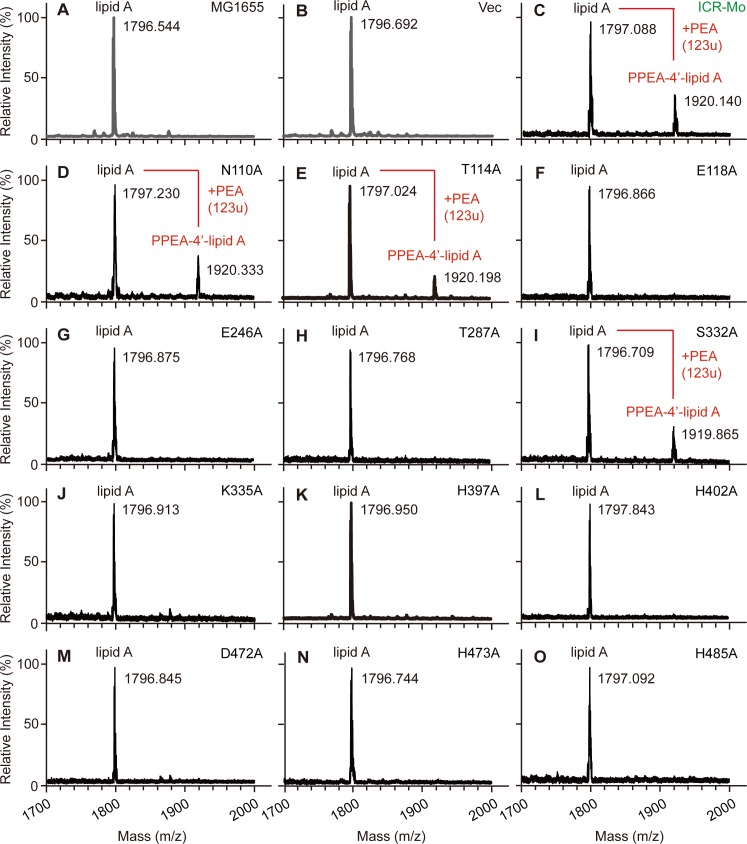
MALDI-TOF MS identification of LPS-lipid A structure suggests different roles of putative cavity-forming sites in catalysis mechanism of ICR-Mo (AXE82_07515). MALDI-TOF MS profile of the LPS-lipid A pool isolated from the two negative controls, *E*. *coli* strain MG1655 alone (**A**) and with the empty vector pBAD24 (**B**). **C.** Appearance of a unique peak of the mono-modified lipid A, PPEA-1(or 4’)-lipid A, in the positive control, MG1655 strain carrying the wild-type of ICR-Mo (AXE82_07515). The two mutations of N110A (**D**) and T114A (**E**) failed to fully inactivate the enzymatic activity of ICR-Mo in that the modified peak, PPEA-lipid A is present. MALDI-TOF-MS analyses suggests that the three mutations of E118A (**F**), E246A (**G**) and T287A (**H**) impairs the function of ICR-Mo. The point-mutant (S332A) of ICR-Mo still possesses partial activity of catalyzing the transfer of PPEA to the 1(4’)-phosphate group of lipid A moieties. The six point-mutants of ICR-Mo are consistently inactive in the enzymatic activity of PEA transferase *in vivo*, including K335A (**J**), H397A (**K**), H402A (**L**), D472A (**M**), H473 (**N**) and H485A (**O**), respectively. The MS peak of lipid A species in *E*. *coli* is shown at m/z of 1796.744~1797.843, whereas its modified form appears at m/z of 1919.865~1920.457, when functional (even partial active) versions of ICR-Mo (AXE82_07515) are present in *E*. *coli*.

In general, the variation in enzymatic activities of ICR-Mo mutants was further verified by structural analyses of LPS-lipid A moieties in a physiological context (**Fig 5**). As expected, MALDI-TOF MS analysis reveals that a unique peak of PPEA-1(or 4’)-lipid A (m/z, 1920.140) is present in the *E*. *coli* expressing the wild-type ICR-Mo (**Fig 5C**), relative to the recipient strain MG1655 with a single peak of lipid A [m/z, 1796.782~1796.946] (**Fig 5A** and **5B**). Consistent with the observations of the TLC experiments, 9 of 12 ICR-Mo point-mutants cannot modify lipid A species *in vivo* since only a single peak of lipid A is detected (**Fig 5F–5H** and **5J–5O**). In contrast, the remaining 3 mutants seem to retain partial abilities of PEA transfer, in that an extra-peak of PPEA-4’-lipid A [m/z, 1919.865~1920.457] is produced in addition to the lipid A [1796.709~1797.230] (**Fig 5D, 5E** and **5I**). Of note, as for the ICR-Mo(T114A) mutant retaining partial activity *in vivo* (**Figs 4C** and **5E**), no enzymatic activity *in vitro* is detected (**S8B Fig**). This could be partially attributed to the lack of sufficient sensitivity of TLC assays in this case. Evidently, this highlights the importance of cavity-forming residues in ICR-Mo biochemistry.

### Domain-swapping analyses of ICR-Mo

**Fig 6:**
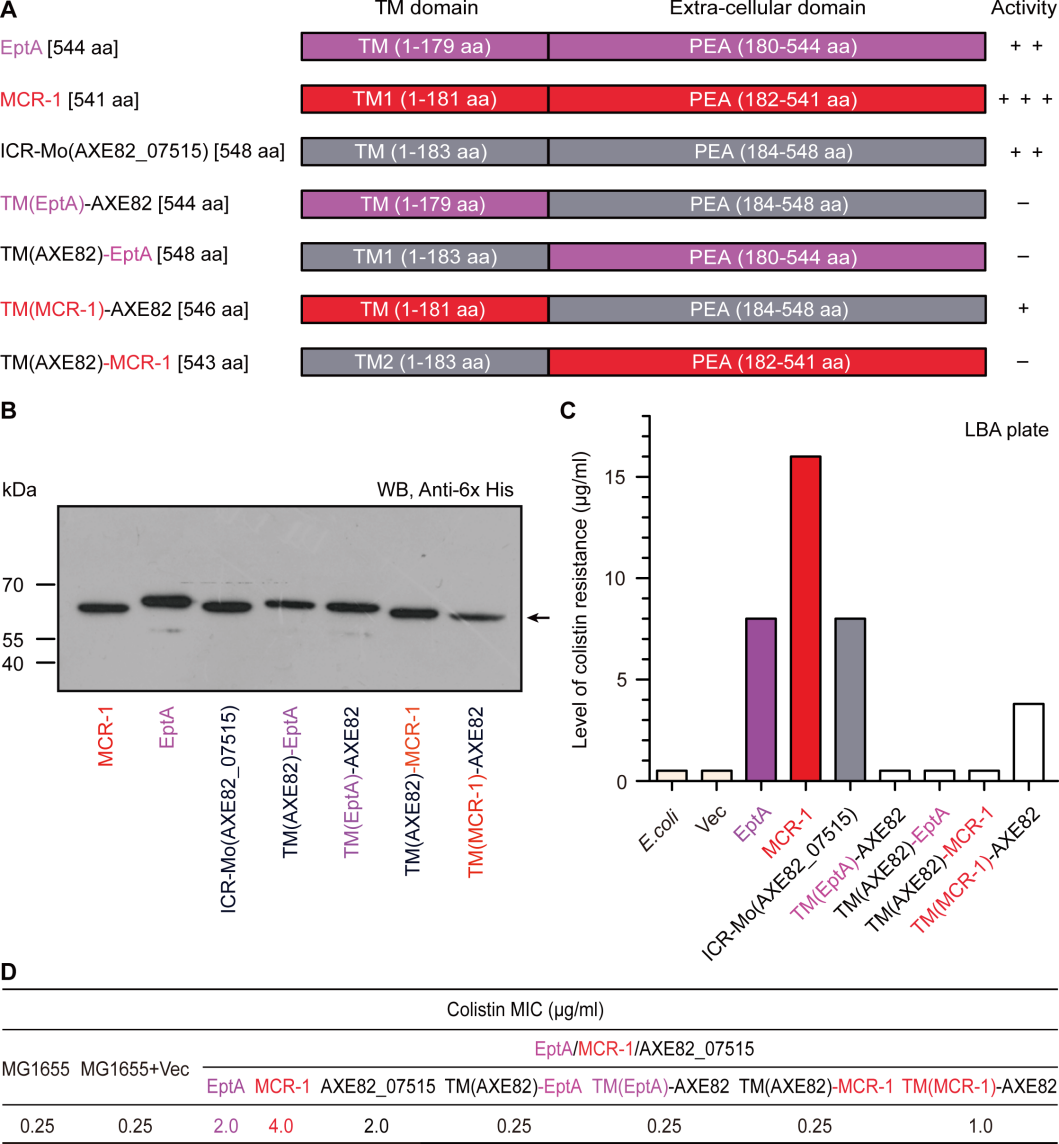
Domain-swapping analyses of ICR-Mo. **A.** Schematic illustration for domain-swapping designing amongst the three transmembrane enzymes EptA, MCR-1 and ICR-Mo (AXE82_07515). **B.** Use of Western blotting to detect the expression of *icr-Mo* and its mosaic versions. **C.** Functional evaluation of ICR-Mo derivatives in ability of conferring appreciable growth of *E*. *coli* on LBA plates with varied levels of colistin. **D.** Measurement of colistin MIC of *E*. *coli* strains expressing ICR-Mo and its hybrid derivatives.

Phylogenetically, ICR-Mo is distinct from EptA and MCR-1/2. This prompted us to probe the possible association of the two modules of MCR-like enzymes [transmembrane region (TM) and PEA transferase domain] with respect to their functional evolution (**Fig 1**). As described with MCR-2 [22], we adopted the method of domain swapping to engineer a collection of hybrid versions of the *icr-Mo* gene (**Fig 6A**). In addition to three parental proteins (EptA, MCR-1 and ICR-Mo (AXE82_07515)), four hybrid proteins engineered here include i) TM(EptA)-AXE82, a modified ICR-Mo carrying TM region of EptA; ii) TM(AXE82)-EptA, a derivative of EptA whose TM domain is replaced with its ICR-Mo counterpart; iii) TM(MCR-1)-AXE82, a modified version of ICR-Mo containing the TM domain of MCR-1; and iv) TM(AXE82)-MCR-1, a hybrid derivative of MCR-1 carrying the TM region of ICR-Mo (**Fig 6A**). Western blotting analyses prove that all the MCR-like enzymes (ICR-Mo and its domain-swapped derivatives) express pretty well in *E*. *coli* (**Fig 6B**). Subsequently, they were subjected to functional evaluation under both *in vitro* and *in vivo* conditions (**S9–S11 Figs**). Following an MS-based confirmation of their polypeptide sequence identity (**S9**and **S10 Figs**), circular dichroism (CD) experiments reveal that all the hybrid derivatives of ICR-Mo consistently display typical CD spectra of being rich in α-helices (**S11D–S11G Fig** and **S3 Table**), almost identical to that of MCR-1 (**S11A Fig**), EptA (**S11B Fig**) and ICR-Mo (**S11C Fig**). As observed with soluble domains of both MCR-1 [40–43, 45] and MCR-2 [46], the result of ICP/MS (inductively coupled plasma mass spectrometry) reveals that zinc is specifically occupied with ICR-Mo and its derivatives (**S12A Fig**), and the relative ratio of zinc ion to protein is calculated to be around 1:1 (**S12B Fig**).

**Fig 7:**
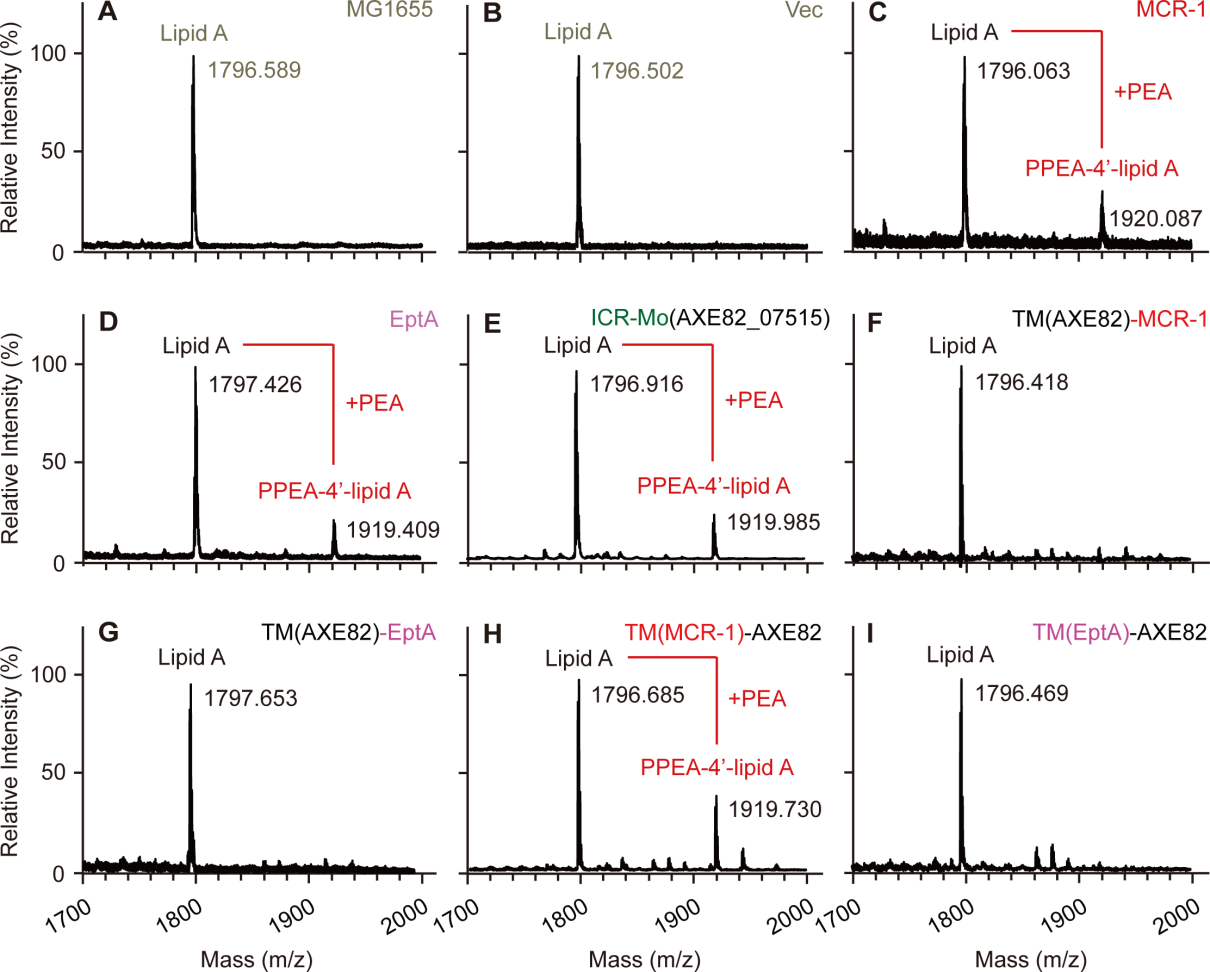
Use of MALDI-TOF mass spectrometry to evaluate physiological role of chimeric versions of ICR-Mo/EptA/MCR-1 in structural modifications of LPS-lipid A species in *E*. *coli*. MALDI-TOF MS suggests a single peak of intact LPS-lipid A species in the *E*. *coli* alone (**A**) or carrying the empty vector pBAD24 (**B**). An additional unique MS peak of the PEA-added (lipid A-4’-PEA) appears in the *E*. *coli* strains expressing MCR-1 (**C**), EptA (**D**) and ICR-Mo (**E**). The three hybrid enzymes [TM(AXE82)-MCR-1 (**F**), TM(AXE82)-EptA (**G**) and TM(EptA)-AXE82 (**I**)] have no detectable activities in transfer of PEA moiety to the 1(4’)-phosphate position of lipid A GlcN moieties. **H.** The mosaic enzyme of TM(MCR-1)-AXE82 exhibits partially enzymatic activity in the formation of lipid A-4’-PPEA from lipid A. The MS spectra (in Panels A and B) functioned as negative controls, whereas the positive controls appeared (in Panels C, D and E). The MS peak of lipid A species in *E*. *coli* is shown at m/z of 1796.063~1797.653, whereas its modified form, PPEA-1(or 4’)-lipid A is detected at m/z 1919.409~1920.087.

Assays of antibiotic resistance on LBA plates show that i) 3 of 4 chimeric versions are inactive; and the only one variant, TM(MCR-1)-AXE82, has partial activity to allowing the recipient *E*. *coli* to grow on the condition with up to 4 μg/ml colistin (**Fig 6C**), less than those of its parental versions MCR-1 (16 μg/ml colistin) and ICR-Mo (8 μg/ml colistin) (**Fig 6C**). Similar scenarios were seen in the measurement of colistin MIC (**Fig 6D**). In addition, MALDI-TOF MS analyses of lipid A species demonstrated that like the wild-type MCR-like proteins (**Fig 7C–7E**), TM(MCR-1)-AXE82 is the only one hybrid derivative of ICR-Mo having a role in the transfer of the PEA moiety from PE to the acceptor LPS-lipid A (**Fig 7H**). In contrast, the other 3 derivatives (**Fig 7G–7I**), have no detectable activities similar to those of negative controls (**Fig 7A** and **7B**). Evidently, MS-based visualization of altered structures of lipid A moieties agrees with scenarios observed in the both enzymatic tests and colistin resistance assays (**Fig 6**). In summary, these data suggest that protein evolution has created a gradient with respect to the acquisition/differentiation of antibiotic resistance amongst EptA, ICR-Mo and MCR-1/2 to some extent.

### ICR-Mo quenches hydroxyl radical death pathway

**Fig 8:**
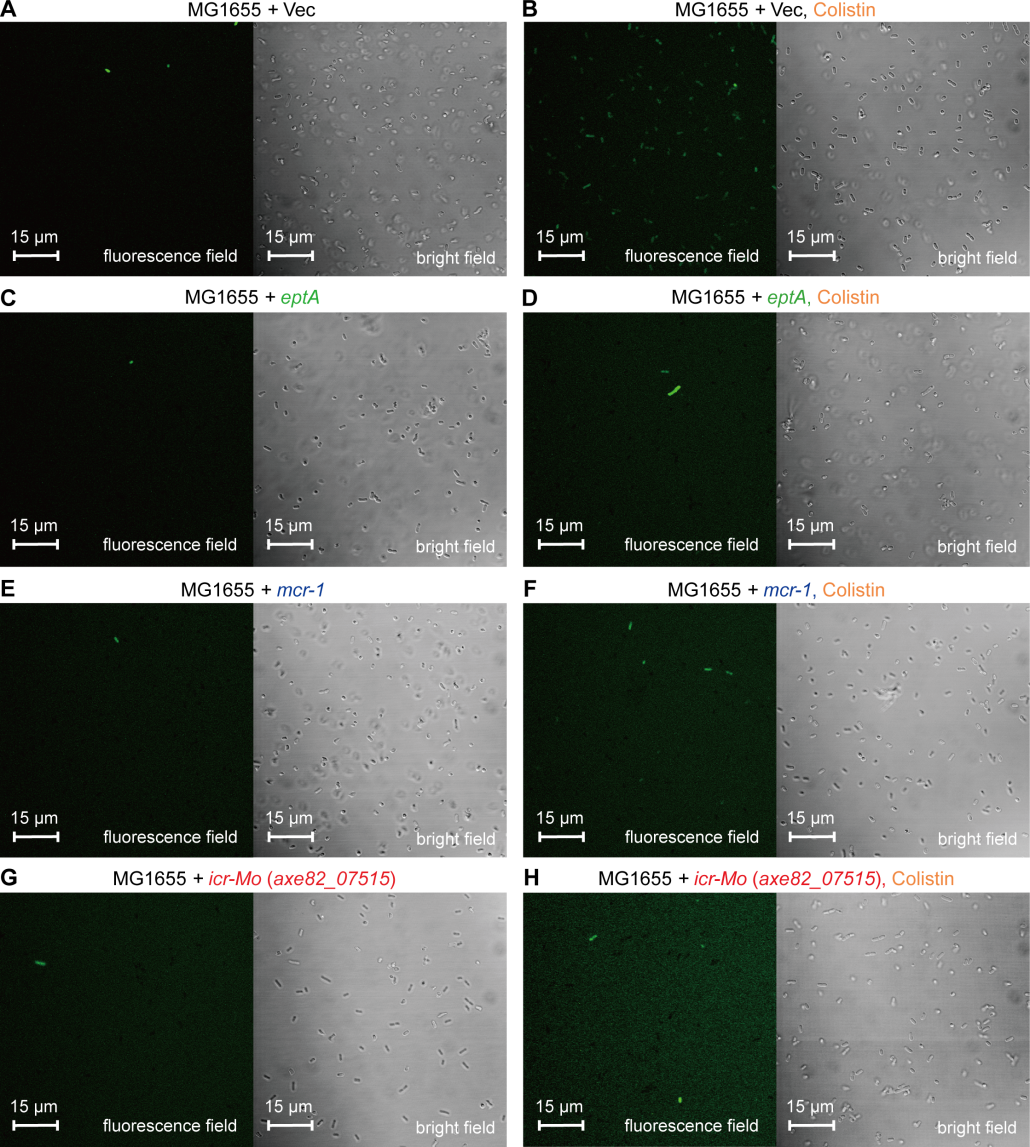
Colistin-stimulated ROS production is significantly impaired by the presence of colistin resistance-conferring proteins (EptA/MCR-1/ICR-Mo). **A & B** Accumulation of hydrogen peroxide is boosted by colistin stress in *E*. *coli*. **C & D** The presence of plasmid-borne EptA inhibits colistin-induced production of hydrogen peroxide in *E*. *coli*. **E & F** Colistin-triggered production of hydrogen peroxide is decreased upon the expression of MCR-1 in *E*. *coli*. **G & H** The expression of *icr-Mo* greatly impairs colistin-promoted accumulation of hydrogen peroxide in *E*. *coli*. The intra-cellular ROS level was detected with an oxidant-sensitive dye, DCFH2-DA. The fluorescent product of DCF was generated due to the oxidation of the dye by hydrogen peroxide. The fluorescence intensity was quantified with a Zeiss LSM 510 Meta confocal laser scanning microscope (100x oil immersion objective). The hydrogen peroxide produced is denoted in green.

**Fig 9:**
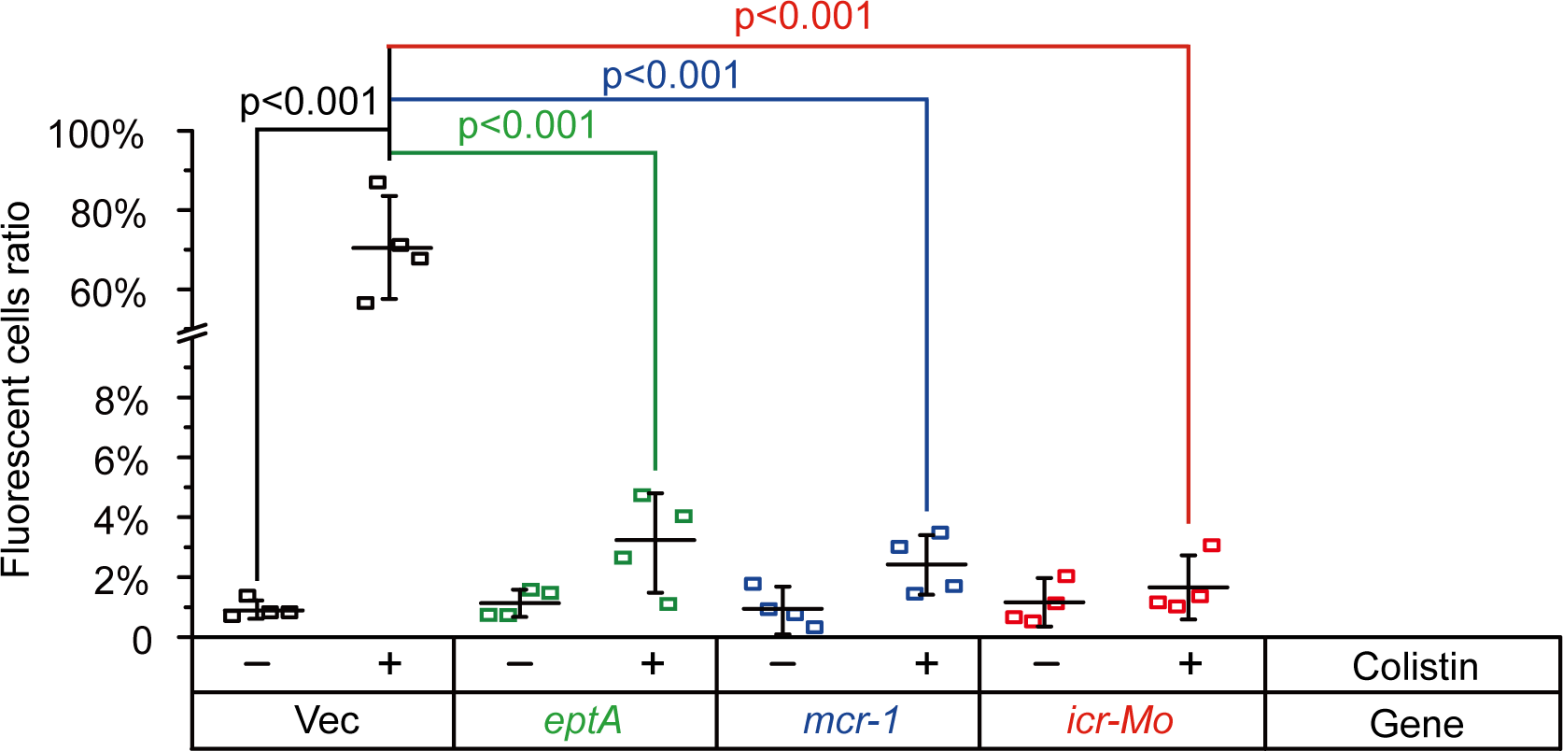
Relative ratio of colistin-induced ROS levels in *E*. *coli* carrying *icr-Mo* (*mcr-1* and/or *eptA*). Ratio of fluorescent cells was obtained by counting the number of cells with/without fluorescence (illustrated in **Fig 8**). In every group, over 500 cells counted from 4 individual photographs. The data was assessed using one-way analysis of variance (ANOVA) followed by Tukey–Kramer multiple comparisons post hoc test.

**Fig 10:**
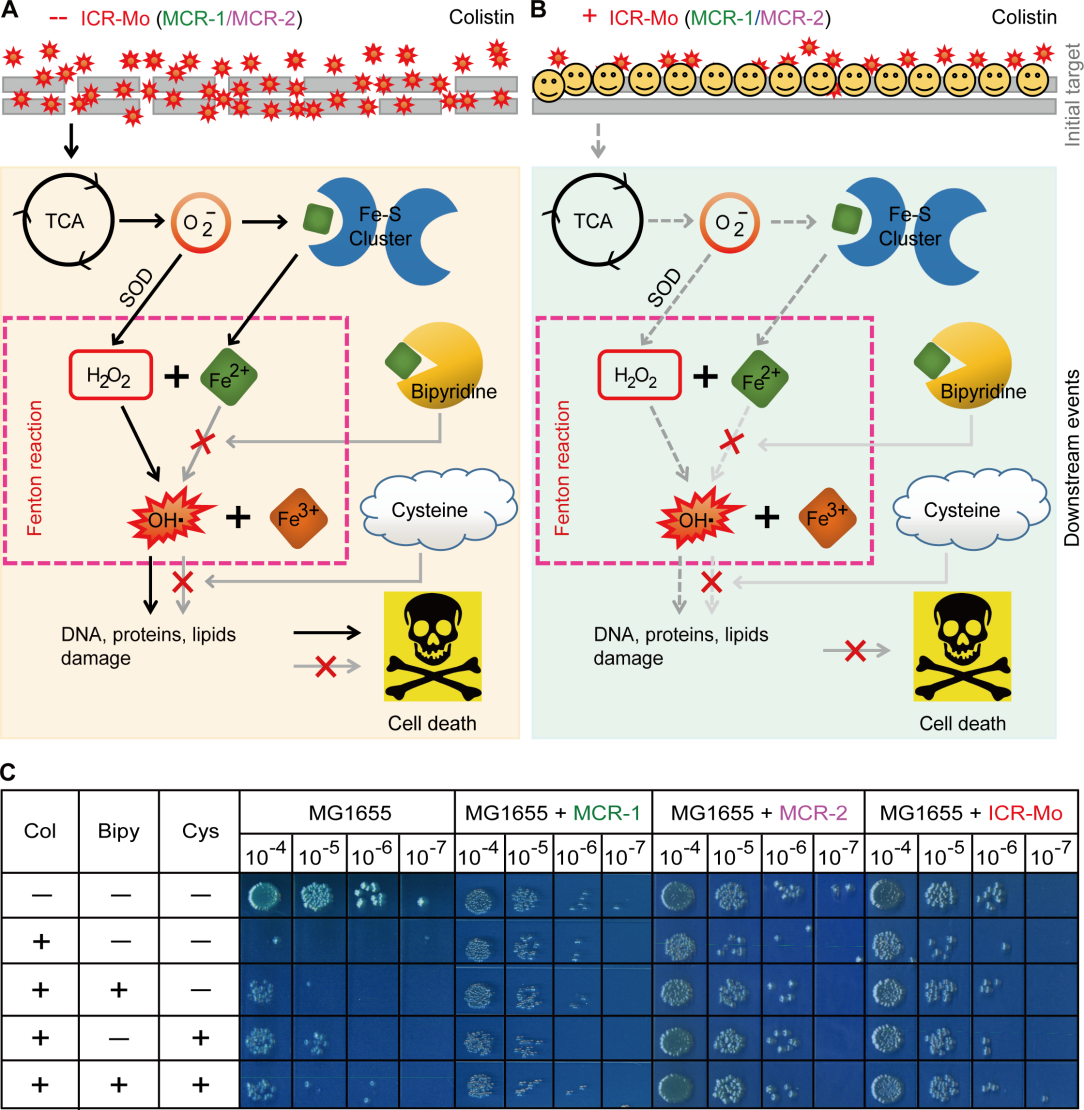
A working model that ICR-Mo stops colistin-induced hydroxyl radical killing in *E*. *coli*. **A.** Scheme for ROS production triggered by colistin in *E*. *coli*. **B.** Impairment of colistin-induced ROS formation in *icr-Mo*-bearing *E*. *coli*. **C.** Chemical rescue experiments reveal that a Fenton reaction is involved in the colistin-activated hydroxyl radical killing pathway in *E*. *coli*. The LPS-lipid A moiety refers to an initial target for colistin treatment. Bipyridine is a well-studied ferric chelator, and L-cysteine is the ROS scavenger.

Successful killing and eradication of the Gram-negative bacteria by polymyxins is determined by two critical factors: i) Efficient binding/entry to the initial target, bacterial LPS-lipid A [9]; ii) Activation of a downstream hydroxyl radical death pathway [47, 48]. The aforementioned results have proved that ICR-Mo can modify the lipid A moieties, whereas it is unclear as to whether or not it influences the downstream event. Therefore, we integrated two different methods [confocal microscopy (**Figs 8**and **9**) and chemical rescue assay (**Fig 10**)] to address this question. First, an oxidant-sensitive dye DCFH2_DA (2′,7′-dichlorodihydrofluorescein diacetate) was applied in monitoring intra-cellular H_2_O_2_ species in *E*. *coli* with/without ICR-Mo (or EptA/MCR-1) (**Fig 8**). As expected, the treatment with colistin boosts ROS production in the *E*. *coli* MG1655 (**Fig 8A** and **8B**). However, expression of *icr-Mo* alleviates colistin-triggered ROS formation regardless of colistin treatment (**Figs 8G** and **9**). This is in agreement with observations in *E*. *coli* strains expressing either EptA (**Fig 8C** and **8D**) or MCR-1 (**Fig 8E** and **8F**).

In chemical rescue trials, the presence of a ferric chelator, bipyridine, significantly bypasses cell death of the *icr-Mo*-negative *E*. *coli* with that are subjected to treatment with colistin (**Fig 10A** and **10C**). It seems likely that a Fenton reaction is involved in the formation of free hydroxyl radicals (**Fig 10A**). Similarly, the presence of the ROS scavenger, L-cysteine alone [or accomplished with bipyridine] also greatly improves bacterial survival of *E*. *coli* under conditions of colistin treatment (**Fig 10A** and **10C**). Intriguingly, the presence of *icr-Mo* (or *eptA/mcr-1*) prevents the recipient *E*. *coli* from entering into the hydroxy radical death pathway in *E*. *coli* (**Fig 10B** and **10C**). Of note, this impact is independent of presence of pyridine (and/or L-cysteine). Thus, we believe that ICR-Mo (EptA/MCR-1) modifies the bacterial LPS-covered membrane, prevents efficient entry of polymyxin into cells, quenches/alleviates ROS production *in vivo*, and consequently bypasses antibiotic killing by colistin (**Fig 10**).

## Discussion

Polymyxin is a paradigm for cationic antimicrobial polypeptides (CAMP). Physical basis for CAMP-type resistance is due to structural alteration of bacterial LPS, leading to a reduction of the net negative charge of the cell surface. In total, three types of chemical modifications of lipid A species are involved in surface remodeling of Gram-negative bacterium. Among them, the most prevalent form refers to the modification of the LPS-lipid A with an addition of the cationic sugar 4-amino-4-deoxy-l-arabinose [49–51]; Second, the transfer of PEA to the 1(or 4’)-phosphate position of lipid A moieties [16, 17, 51–54]; Third, the glycine attachment to LPS-lipid A GlcN moieties by a tripartite system [Vc1577 (AlmG), Vc1578 (AlmF), and Vc1579 (AlmE)] in *Vibrio cholerae* [55]. It is known that rare cases of an intrinsic colistin resistance are generated by spontaneous point-mutations of chromosomal genes, especially two-component systems of PhoB/Q [53, 56] and PmrA/B [17, 52–54]. In addition, the EptA enzyme of the *Neisseria* species, which is a representative member of PEA lipid A transferase family, also contributes to the generation of an intrinsic resistance to colistin [16]. In contrast, the discovery of MCR-like determinants belonging to PEA lipid A transferases constitutes a new mechanism for the transferability of polymyxin resistance [10, 14, 21, 22, 24–26]. It seems more worrisome that global transmission of *mcr-1* genetic determinant on diversified plasmids raises significant challenge to clinical therapy and public health [11, 57, 58]. Therefore, it is essential to understand the evolution of *mcr-1*-like variants.

Very recently, *Moraxella* species have been proposed as a reservoir for *mcr-1/2* genetic determinants [30–32]. Our phylogenetic analyses showed that the MCR-1/2 family is much more closely related to the *Moraxella* family of MCR-like genes than either Sulfatases or other non MCR-like genes. In fact, the *pap2* gene originally found next to *mcr-1* on plasmids, has also been detected on the chromosome next to *mcr-1* (and its genetic variants) in certain other species of *Moraxella* [30]. Given the fact that i) both *M*. *pluranimalium* [59] and *M*. *porci* [60] colonize in pigs; and ii) pigs are major host reservoirs for *mcr-1*-harboring Enterobacteriaceae [10], it is ecologically reasonable that genetic exchange of *mcr-1/2*-like determinants between *Moraxella* species and Enterobacteriaceae like *E*. *coli* occur. This leads us to believe that evaluating the chromosomally-encoded genetic determinants of colistin resistance in *Moraxella* might further our understanding of evolution in the MCR1/2 family. The data we report here presents a biochemical and physiological understanding of ICR-Mo, found on the chromosome of *Moraxella osloensis*. Relative to MCR-1, ICR-Mo confers a lower level of resistance to colistin to a recipient *E*. *coli* strain (**S6 Fig**), which argues the possibility that *mcr*-like genes are in either a developing or degenerating state [61]. Of note, any direct evidence of ICR-Mc [33] or ICR-Mo conferring resistance to colistin in native *Moraxella* strains is also lacking. The two domains (TM region and extra-domain of PEA transferase) of MCR-1 and MCR-2 are functionally-exchangeable [22], while those in ICR-Mo and MCR-1 cannot be fully switched (**Fig 6**). This indicates a fundamental difference between these two proteins that further validates their phylogenetic placement. Structural and functional characterization of ICR-Mo facilitates the identification of an important PE substrate cavity, which is essential for its enzymatic activity and for the conferring a phenotypic resistance to colistin (**Figs 3**and **4**). In light of its similarity to those of EptA and MCR-1 (**Figs 3**and **4**), this hints an evolutionarily-conserved mechanism for intrinsic and transferable colistin resistance. In fact, this is in close agreement with observations made from a very recent X-ray crystal structure of an intrinsic colistin resistance gene product, ICR-Mc, in *Moraxella catarrhalis* [33]. Moreover, ICR-Mo displays similar abilities in both modifying lipid A structure (**Fig 2**) and quenching the production of ROS *in vivo* (**Figs 8**and **9**), when compared with that of MCR-1. Intriguingly, it seems likely that a putative “ping-pong” mechanism is shared amongst these MCR-like enzymes (**S1 Fig** and **Fig 2**). It was thought that the ancestral source of colistin resistance might be from proteins containing sulfatase/hydrolase domains. These domains possess the same catalytic architecture as PEA transferases. Further, polymyxin is naturally produced by (and originally isolated in) certain members of the *Paenibacillus* family as a secondary metabolite. However, based on our biochemical and phylogenetic data it is tough to conclude whether the members of the non MCR-like family (comprising MCR-3/4), which cluster closely with the Sulfatase family, are in a state of development or degeneration of colistin resistance. Thus, we simply propose that ICR-Mo and the other members of the *Moraxella* family have a same ancestor as the MCR-1/2 family does, providing a current source of genetic variation.

In response to the emergence of MCR-1 colistin resistance in the very late of 2015 [10], Chinese agricultural government has taken on a more active role in preventing the further spread of MCR-1 colistin resistance by formally banning the use of colistin as a growth booster in China in early 2017. Moreover, it is also important to reconsider the clinical use of colistin as a final line of refuge against lethal infections with carbapenem-resistant superbugs. Our findings represent a full mechanistic understanding of ICR-Mo, a representative member of a family of chromosomal relatives to transferable MCR-1 colistin resistance. It might provide molecular basis for the rational development of small molecules targeting the reversal of MCR-like resistance to colistin, a last-resort antibiotic.

## Materials and methods

### Strains, plasmids and growth conditions

All the strains used here are derivatives of *E*. *coli* MG1655 (**S1 Table**). As described with *mcr-2* [22], the full-length *icr-Mo* (AXE82_07515) was synthesized *in vitro*. To generate hybrid versions of *icr-Mo* and *mcr-1* (and/or *eptA*), overlapping PCR experiments were conducted [22]. The point-mutants of *icr-Mo* were produced using the Mut Express II Fast Mutagenesis Kit V2 (Vazyme Biotech Co., Ltd) with suitable primers (**S1 Table**). The pET21a expression vector was used for protein production, and arabinose-inducible plasmid pBAD24 was utilized for functional complementation (**S1 Table**). All the constructs were confirmed with direct DNA sequencing. Luria-Bertani (LB) broth (either liquid culture or solid agar plates) was applied and appropriate antibiotics such as ampicillin and colistin were supplied.

### Assays for colistin susceptibility

The minimum inhibitory concentration (MIC) of colistin was determined using a liquid broth dilution test as recommended by EUCAST with Cation-adjusted Mueller-Hinton Broth (CAMHB)[22]. When necessary, 0.2% arabinose was added into CAMHB media to induce the expression of pBAD24-borne *icr-Mo* and its derivatives in *E*. *coli*. Also, the viabilities of *E*. *coli* carrying *icr-Mo* and its derivatives were judged with solid LBA broth dilution test [14, 62, 63].

### Expression, purification and identification of transmembrane enzyme ICR-Mo

As descried with MCR-2 [22] with little change, ICR-Mo and its chimeric versions were overexpressed. The membrane fraction containing the protein of interest was solubilized in buffer B (20 mM Tris-HCl [pH 8.0], 100 mM NaCl, 5% glycerol, 1% DDM (M/V)) and then centrifuged at 38000 r.p.m for 1.5 h at 4°C. The resultant supernatant was subjected to affinity purification of protein with pre-equilibrated Ni-NTA agarose beads. The expected integral membrane proteins of ICR-Mo and its derivatives were eluted using an elution buffer (20 mM Tris-HCl [pH 8.0], 100 mM NaCl, 100 mM imidazole, 5% glycerol, 0.03% DDM (M/V)) [14, 22]. Following determination of the purified protein with 12% SDS-PAGE, the protein band with expected size was cut from gels and subjected to MS-based identification. The data of acquired polypeptides was confirmed by BLAST against NCBI NR database.

### Experiments of circular dichroism and inductively coupled plasma mass spectrometry

Circular dichroism (CD) tests were conducted to characterize secondary structures of ICR-Mo and its chimeric variants in the Tris buffer [20 mM Tris-HCl [pH 8.0], 100 mM NaCl, 5% glycerol, 0.03% DDM]. The CD spectra were recorded on a Jasco Model J-1500 spectrometer (Jasco Corp., Tokyo, Japan) through continuous wavelength scanning (in triplicate) from 190 to 240 nm at a scan rate of 50 nm/min [64] and smoothed with a Savitsky-Golay filter [65]. CD spectra were analyzed using SELCON3 program (protein basis set 10) in the CDPro software package (http://lamar.colostate.edu/~sreeram/CDPro/), developed at the Department of Biochemistry and molecular Biology of Colorado State University [66]. The different percentages were measured, which corresponded to four major types of secondary structure motifs (α-helices, β-sheet, η-turn and coils) [67].

To further probe whether or not zinc ions are occupied in ICR-Mo and their derivatives, inductively coupled plasma mass spectrometry (ICP-MS) was performed. The protein samples (~0.2 mg/ml) were subjected to the NexION 300X ICP-MS instrument (PerkinElmer, USA) with helium as carrier gas [68]. The mass-to-charge ratio (m/z) was measured in the mode of kinetic energy discrimination (KED) mode. In total, seven proteins were examined, which include 3 parental enzymes (ICR-Mo [AXE82_07515], EptA and MCR-1) and 4 hybrid versions (namely TM(AXE82)-EptA, TM(EptA)-AXE82, TM(AXE82)-MCR-1 and TM(MCR-1)-AXE82).

### *In Vitro* enzymatic assays

As described with EptA by Anandan *et al*. [34], we tested the enzymatic activity of ICR-Mo *in vitro*. In this enzymatic reaction system, the fluorescent substrate, is abbreviated as NBD-PEA from 1-acyl-2-{12-[(7-nitro-2-1,3-benzoxadiazol-4-yl) amino] dodecanoyl}-sn-glycero-3-phosphoethanolamine (Avanti Lipids, USA). The reaction system (50 μl in total) consists of 50 mM HEPES (pH 7.50), 100 mM NaCl, 0.03% of DDM, 0.2 mM NBD-PEA and 40 μM ICR-Mo [MCR-1/MCR-2 and derivatives]. The reaction proceeded at 25°C for around 24 hrs [36, 37]. Thin layer chromatography (TLC) was performed to detect the presence of the NBD-glycerol product hydrolyzed from its substrate NBD-glycerol-3-PEA. Of note, fluorescence signal of NBD-glycerol-3-PEA (and/or NBD-glycerol) separated with TLC plates was visualized by Epi blue light (455–485 nm) and a corresponding filter of a ChemiDoc MP imaging system (Biorad, CA, USA) [34].

The identity of both NBD-glycerol-3-PEA and NBD-glycerol was determined by Liquid Chromatography Mass Spectrometry(LC/MS) system (Agilent technologies 6460 Triple Quad LC/MS) [69]. Mass spectrometry was coupled with electrospray ionization (ESI) source, in which neutral loss ion (m/z 141) mode was set for scanning of the positive ion. The samples were eluted with the solution of methanol/0.1% methanoic acid (95:5) at 0.3 ml/min and separated with an analytical chromatographic column of Zorbax SB C18 (2.1*50 mm, 3.5 μm)

### Extraction, purification and identification of LPS-lipid A

LPS-lipid A was extracted as described by Liu *et al*. [70]. Following separation with SDS-PAGE (10%), the purity of LPS specimens was judged with silver staining [36, 37]. The lipid A species that satisfied the purity criteria were then subjected to structural identification with MALDI-TOF-MS (Bruker, ultrafleXtreme) in negative ion mode with the linear detector [70].

### Confocal microscopy

Mid-log phase cultures of *E*. *coli* strains were used in the challenge by colistin (4 μg/ml, 0.5 h). The oxidant sensor dye DCFH2-DA (i.e., 2′,7′-dichlorodihydrofluorescein diacetate, Sigma) was utilized to detect intra-cellular accumulation of reactive oxygen species (ROS). The fluorescent dichlorofluorescein (DCF), the oxidation product of DCFH2-DA, was visualized using a Zeiss LSM 510 Meta confocal laser scanning microscope [71]. Totally, four types of *E*. *coli* strains used here correspond to FYJ796 [MG1655 with empty vector] which served as the negative control, FYJ832 [MG1655 carrying pBAD24::*eptA*], FYJ795 [MG1655 carrying pBAD24::*mcr-1*] and FYJ968 [MG1655 with pBAD24::*axe82_07515*], respectively (**S1 Table**).

### Chemical rescue tests

Using two ROS inhibitors: bipyridine (a ferric chelator) and L-cysteine (a ROS scavenger), chemical rescue experiments were carried out as described by Collins and coauthors [72, 73] with minor modifications. Following different challenges, bacterial viability of *E*. *coli* strains (with or without *icr-Mo* (or *mcr-1/2*)) was recorded. The four different kinds of challenges employed here were i) colistin alone; ii) colistin combined with 2,2’-dipyridine [74]; iii) colistin mixed with L-cysteine [75] and iv) the mixture consisting of colistin, 2,2’-dipyridine [74] and L-cysteine [75]. In this assay, compounds were supplemented as follows: 20 μg/ml for colistin (Sigma), 500 μM for 2,2’-dipyridine (Sangon Biotech) and 10 mM for L-cysteine (Sangon Biotech). After 30 mins of incubation at 37°C, cultures were serially diluted. The selected dilutions (10^−4^~10^−7^) were dropped onto LB agar plates (5 μl each). Colony forming units (CFU) were enumerated [76].

### Bioinformatics, structural modelling and molecular docking

Sequence alignment of ICR-Mo (AXE82_07515) with its paralogues was conducted with Clustal Omega (http://www.ebi.ac.uk/Tools/msa/clustalo/) and processed via the program ESPript 3.0 (http://espript.ibcp.fr/ESPript/cgi-bin/ESPript.cgi)[77]. The trans-membrane region of ICR-Mo was predicted using TMHMM server v2.0 (http://www.cbs.dtu.dk/services/TMHMM/). Using the *N*. *meningitis* EptA (PDB accession number, 5FGN) as structural template [34], architecture of ICR-Mo was modeled via Swiss-Model [35]. The value of GMQE (Global Model Quality Estimation) is 0.68 and the score of QMEAN (which provides a global and local absolute quality estimate on the modeled structure [78]) is -3.62. Thus, it suggests a reliable qualified structural prediction. The ready-to-dock chemical structure of PE (ID: ZINC32837871) and head group of PE (ID: ZINC02798545) was sampled from ZINC database [79].

The UCSF DOCK 6 software (version 6.7) was applied to predict binding patterns of PE molecule vs EptA and head group of PE to ICR-Mo [80]. LigPlot+ was used to illustrate the diagrams for possible ligand-protein interaction [81]. Concretely, protein structure was processed for molecular docking using UCSF Chimera software [82]. Solvent molecules were removed. Hydrogens were added and charges were assigned using chimera tool Dock Prep. Preferred orientation of PE in EptA was searched in 20 Å space around complexed ligand dodecyl-β-D-maltoside (DDM) in 5FGN. A set of spheres located in 20 Å space around DDM was calculated by the use of the program Sphere_select. The accessory program GRID in DOCK6 software package was used to compute van der Waals potential grid and electrostatic potential grid for energy scoring. Given that PE molecule contains many rotatable bonds and is thus more flexible, anchor-and-grow algorithm was set for conformational search in docking studies. This type of flexible ligand docking allows the ligand to structurally rearrange in response to the receptor.

### Phylogenetic analysis

The nucleotide sequence of AXE82_07515 (designated as *icr-Mo*) from *M*. *osloensis* was used as a query to perform a nucleotide BLAST with options enabled to exclude models and uncultured environmental samples. The blastn algorithm parameters were modified to display 1000 target sequences with the query word size set to 7, allowing it to return ‘somewhat similar sequences’ to the query. All sequences with a minimum of 30% identity and greater than 70% query coverage were selected and exported. In addition, members of the distantly related Sulfatase family, if not identified in the blast search, were included. Unique nucleotide sequences were identified using Uniqueseq (https://www.ncbi.nlm.nih.gov/CBBresearch/Spouge/html_ncbi/html/fasta/uniqueseq.cgi) and aligned using MUSCLE (https://www.ebi.ac.uk/Tools/msa/muscle/). A total of 54 unique nucleotide sequences were utilized for the subsequent phylogenetic analysis. The 5’ end of the aligned sequences was trimmed to improve conservancy and then utilized for phylogenetic analysis. jModeltest (via MEGA 7 [83]) was used to identify the best-fit nucleotide substitution model. Using MEGA 7 [83], the best model obtained from before was used to generate a maximum-likelihood tree with 1000 bootstrap replicates.

Initial tree(s) for the heuristic search were obtained automatically in MEGA 7 by applying Neighbor-Joining and BioNJ algorithms [84] to a matrix of pairwise distances estimated using the Maximum Composite Likelihood (MCL) approach, and then selecting the topology with superior log likelihood value. A GTR model with discrete Gamma distribution and Invariant sites [85] was used to model evolutionary rate differences among sites (5 categories (+G, parameter = 1.1625)).

## Supporting information

S1 Table Bacteria and plasmids used in this study. (DOCX)

S2 Table Primers used in this study. (DOCX)

S3 Table Comparative analyses of secondary structure motifs among the EptA, MCR-1, ICR-Mo and their derivatives. (DOC)

S1 Fig A scheme for the enzymatic reactions catalyzed by ICR-Mo (AXE82_07515). **A.** Chemical reaction proposed for the transfer of PEA to lipid A by ICR-Mo. ICR-Mo catalyzes the addition of PEA to the 1 or 4’ -position of lipid A moiety anchored on lipopolysaccharides, in which final products referred to PEA-4’-lipid A and diacylglycerol. The chemical structures of molecules are depicted with ChemDraw software. **B.** First-half reaction proposed for the ICR-Mo catalysis is illustrated with the removal of PEA from PE, giving the final product DG and an adduct of ICR-Mo-bound PEA. **C.** Second-half reaction proposed for ICR-Mo catalysis, which involves the generation of the final product Kdo2-lipid A-1 (or 4’)-PPEA through the transfer of the intermediate product ICR-Mo-bound PEA to the recipient Kdo2-lipid A.
(TIF)

S2 Fig The Z1140 of the *E*. *coli* O157: H7 strain EDL933 (a putative member of the lipid A PEA transferase family) is not functional in the trials of LBA-based colistin susceptibility. The *E*. *coli* MG1655 cultures in mid-log phase were spotted on LBA plates with different levels of colistin (0, 0.5, 1.0, 2.0, 4.0, 8.0, 16.0, and 32.0 μg/ml) and maintained for 20 hrs at 37°C. Prior to bacterial spotting, 0.2% arabinose was mixed into the melted media of LBA to trigger expression of pBAD24-based MCR-like genes as we described [86, 87]. Designations: Vec, the arabinose-inducible pBAD24 vector; TM-MCR-1, a hybrid version of MCR-1 in which the TM region is replaced with the counterpart in Z1140 of the *E*. *coli* O157:H7 strain EDL933; TM1-Z1140, a mosaic derivative of Z1140 carrying the TM domain of MCR-1.Here, it seems likely that Z1140 and its two domain-swapped versions (TM-MCR-1 & TM1-Z1140) have no roles in conferring the colistin-susceptible MG1655 resistance to polymyxin. A representative photograph of three independent assays is given.
(TIF)

S3 Fig Bioinformatic analyses of ICR-Mo (AXE82_07515). **A.** Transmembrane prediction of ICR-Mo. **B.** Sequence comparison of ICR-Mo with EptA and MCR-1/2 homologs. The polypeptide sequences of four enzymes (EptA, ICR-Mo, MCR-1 and MCR-2) were subjected to Clustal Omega (http://www.ebi.ac.uk/Tools/msa/clustalo/) for sequence alignments. The final output is generated with the program ESPript 3.0 (http://espript.ibcp.fr/ESPript/cgi-bin/ESPript.cgi) [22]. The trans-membrane (TM) region was underlined in blue following the prediction with TMHMM server v2.0 (http://www.cbs.dtu.dk/services/TMHMM) and underlined in blue. The five Zn^2+^-interacting residues (E248, T287, H397, D472 and H473) are indicated with green arrows, and the seven PE substrate-binding residues highlighted with red arrows included N110, T114, E118, S332, K335, H402, and H485, respectively. Identical residues are in white letters with red background, similar residues are in red letters with white background, and the varied residues are in black letters. The protein secondary structure was shown in cartoon (on top). Designations: α: α-helix; β: β-sheet; T: Turn; η: coil; PH: Periplasmic-facing helix.
(TIF)

S4 Fig Structural characterization of ICR-Mo. **A.** SDS-PAGE (12%) profile of the purified ICR-Mo protein. **B.** Scheme for the ICR-Mo topology. **C.** Overall architecture of the full-length ICR-Mo integral membrane protein. The overall structure of ICR-Mo (AXE82_07515) in ribbon was modeled using the *Neisseria meningitis* EptA (PDB: 5FGN) as structural template. The catalytic domain is illustrated in blue, the TM region is highlighted in magenta and the two helices (PH2 and PH2’) are in light grey. The rectangle with light peach background refers to the layer of inner-membrane. The red sphere denotes zinc ion. Designations: TM, Trans-membrane; PH, Periplasmic-facing helices; H, α-helix; S, β-sheet; N, N-terminus; C, C-terminus.
(TIF)

S5 Fig ICR-Mo, a homolog of MCR-1/2 can hydrolyze NBD-glycerol-3-PEA to give NBD-glycerol. **A.** Scheme for ICR-Mo-based hydrolysis reaction of NBD-glycerol-3-PEA into NBD-glycerol. **B.** TLC analyses of the alternative substrate NBD-glycerol-3-PEA and its resultant product NBD-glycerol. Thin layer chromatography (TLC) was performed as Anandan *et al*. [34] described with minor changes. The identities of both NBD-glycerol-3-PEA and NBD-glycerol were validated with LC/MS (seen in **Fig 1**). Minus denotes no addition of the enzyme of EptA/MCR-1/MCR-2/ICR-Mo.
(TIF)

S6 Fig Comparison of bacterial growth of *E*. *coli* strains expressing EptA, MCR-1 or ICR-Mo on the LBA plates supplied with varied levels of colistin. The serial diluted cultures (in mid-log phase) were spotted on LBA plates with various concentrations of colistin (0, 0.5, 1.0, 2.0, 4.0, 8.0, 16.0, and 32.0 μg/ml) and maintained at 37°C for ~20 hrs. Plasmid-borne expression of *icr-Mo* (*axe82_07515*)*/eptA*/*mcr-1* is dependent on the arabinose-inducible expression vector pBAD24 in *E*. *coli* MG1655. 0.2% arabinose is supplied here as an inducer. A representative result of three independent tests is given.
(TIF)

S7 Fig Functional dissection of the PE lipid substrate-recognizable cavity of ICR-Mo (AXE82_07515). **A.** Surface structure of ICR-Mo with the cavity required for entry and binding of PE substrate. **B.** An enlarged illustration for a five residues-containing, Zn^2+^-binding motif. The five residues in Zn^2+^-binding motif of ICR-Mo refers to E248, T287, H397, D472 and H473, respectively. **C.** Surface structure of ICR-Mo in counter-clockwise rotation (30°). **D.** Fine structural illustration of the seven residues-containing motif that is involved in binding of ICR-Mo to PE lipid substrate. The seven residues corresponded to N110, T114, E118, S332, K335, H402 and H485, respectively.
(TIF)

S8 Fig Use of *in vitro* enzymatic assays to evaluate the impact of PE-binding cavity on ICR-Mo catalysis. **A.** SDS-PAGE (12%) profile of the purified ICR-Mo transmembrane enzyme and its 12 derivatives. The estimated mass of ICR-Mo (and its derivatives) is ~60 kDa, indicated with an arrow. **B.** TLC-based analyses of *in vitro* enzymatic activities of ICR-Mo and its 12 point-mutants. The identities of both NBD-glycerol-3-PEA and NBD-glycerol were validated by LC/MS (seen in **Fig 1**). This suggests that only three point-mutants of ICR-Mo (N110A, T114A & S332A) retain partial enzymatic activities to hydrolyze the alternative substrate NBD-glycerol-3-PEA into NBD-glycerol. TLC experiments were conducted as described by Anandan *et al*. [34] with minor modifications. A representative result from three independent trials is given, and this photograph was generated through combining three different TLC plates with limited wells.
(TIF)

S9 Fig MALDI-TOF measurement of molecular mass of ICR-Mo (AXE82_07515) and its derivatives. **A.** MALDI-TOF determination of molecular weight of ICR-Mo protein. **B.** Use of MALDI-TOF to measure molecular mass of an intact hybrid version of ICR-Mo protein, TM(AXE82)-EptA. **C.** Molecular mass of TM(EptA)-AXE82 (a mosaic derivative of AXE82_07515 protein) revealed by MALDI-TOF. **D.** Molecular mass of a hybrid version of ICR-Mo (AXE82_07515) protein, TM(AXE82)-MCR-1. **E.** Molecular weight of TM(MCR-1)-AXE82, a hybrid version of AXE82_07515
(TIF)

S10 Fig MS identification of ICR-Mo (AXE82_07515) and its derivatives. **A.** Protein sequence of *Neisseria gonorrhea* EptA. **B.** Protein sequence of MCR-1. **C.** MS-based identification of ICR-Mo. **D.** MS verification of the chimeric version of ICR-Mo, TM(AXE82)-EptA. **E.** MS determination of a hybrid version of ICR-Mo, TM(AXE82)-MCR-1. **F.** MS-based elucidation of the mosaic version of ICR-Mo, TM1(MCR-1)-AXE82. **G.** MS identification of the hybrid deriivative of ICR-Mo, TM(EptA)-AXE82. The bold letters with yellow background denote the TM regions, and the other letters refer to catalytic domains. The underlined letters correspond to the polypeptides identified by mass spectrometry. Designations: TM(AXE82)-EptA, a derivative of AXE82_07515 whose extracellular region is replaced with its counterpart in EptA; TM(AXE82)-MCR-1, a derivative of AXE82_07515 whose extracellular region is replaced with its counterpart in MCR-1; TM1(MCR-1)-AXE82, a derivative of AXE82_07515 whose TM region is replaced with its counterpart in MCR-1; TM(EptA)-AXE82, a derivative of AXE82_07515 in which TM region is replaced with that of EptA.
(TIF)

S11 Fig Circular dichroism (CD) analyses of ICR-Mo (AXE82_07515) and its chimeric versions. **A.** CD-based visualization of secondary structure of MCR-1 protein. **B.** CD spectrum of EptA protein. **C.** CD assays for ICR-Mo. **D.** CD spectrum of the ICR-Mo derivative, TM(AXE82)-EptA. **E.** CD spectrum of the hybrid version of AXE82_07515, TM(EptA)-AXE82. **F.** CD spectrum of the mosaic version of AXE82_07515, TM(AXE82)-MCR-1. **G.** CD profile of the mosaic version of AXE82_07515, TM(MCR-1)-AXE82. Here, the CD results of EptA/MCR-1/ICR-Mo and their domain-swapped versions indicate they share a similar conformation in protein secondary structures. Abbreviations: CD, Circular dichroism; TM(AXE82)-EptA, a derivative of AXE82_07515 whose extracellular region is replaced with its counterpart in EptA (**Panel B**); TM(EptA)-AXE82, a derivative of AXE82_07515 whose TM region is replaced with its counterpart in EptA (**Panel B**); M(AXE82)-MCR-1, a derivative of AXE82_07515 whose extracellular region is replaced with its counterpart in MCR-1 (**Panel A**); TM1(MCR-1)-AXE82, a derivative of AXE82_07515 whose TM region is replaced with its counterpart in MCR-1 (**Panel A**).
(TIF)

S12 Fig Physical evidence for the presence of zinc ions in the ICR-Mo protein and its derivatives. **A.** Inductively coupled plasma mass spectrometry (ICP/MS)-based detection of Zn^2+^ in ICR-Mo/MCR-1/EptA and the related chimeric derivatives. **B.** Use of ICP/MS to calculate the relative ratio of protein-bound Zn^2+^ to protein. Similar to the observations we recently reported [36, 37], ICP/MS data of ICR-Mo confirms that it might possess zinc ions at the relative ratio of 1:1 (zinc: protein). Indeed, it is also consistent with scenarios seen with the crystal structure of the neissertial EptA [20, 34]. The designations of ICR-Mo derivatives are identical to those of **S11 Fig**.
(TIF)
