# Supplementary material for: Defining ICR-Mo, an intrinsic colistin resistance determinant from Moraxella osloensis
Source: PLoS Genet. 2018 May 14;14(5):e1007389. doi: 10.1371/journal.pgen.1007389 (PMC5983563; doi:10.1371/journal.pgen.1007389)
Supplement: S1 Table — (DOCX) [file pgen.1007389.s001.docx]

**Table S1** Bacteria and plasmids used in this study

| **Strain or plasmids** | **Relevant characteristics** | **Origins** |
| --- | --- | --- |
| **Strains** | | |
| DH5α | A cloning host of *E. coli* | Lab stock |
| MG1655 | A wild type strain of *E. coli* | Lab stock |
| FYJ795 | MG1655 carrying pBAD24*::mcr-1* | Lab stock |
| FYJ796 | MG1655 carrying pBAD24 | Lab stock |
| FYJ832 | MG1655 carrying pBAD24*::eptA* | Lab stock |
| FYJ968 | MG1655 carrying pBAD24*::axe82_07515* | This work |
| FYJ969 | MG1655 carrying pBAD24*::axe82_07515* (E248A) | This work |
| FYJ970 | MG1655 carrying pBAD24*::axe82_07515* (T287A) | This work |
| FYJ971 | MG1655 carrying pBAD24*::axe82_07515* (H397A) | This work |
| FYJ972 | MG1655 carrying pBAD24*::axe82_07515* (D472A) | This work |
| FYJ973 | MG1655 carrying pBAD24*::axe82_07515* (H473A) | This work |
| FYJ974 | MG1655 carrying pBAD24*::axe82_07515* (N110A) | This work |
| FYJ975 | MG1655 carrying pBAD24*::axe82_07515* (T114A) | This work |
| FYJ976 | MG1655 carrying pBAD24*::axe82_07515* (E118A) | This work |
| FYJ977 | MG1655 carrying pBAD24*::axe82_07515* (S332A) | This work |
| FYJ978 | MG1655 carrying pBAD24*::axe82_07515* (K335A) | This work |
| FYJ979 | MG1655 carrying pBAD24*::axe82_07515* (H402A) | This work |
| FYJ980 | MG1655 carrying pBAD24*::axe82_07515* (H485A) | This work |
| FYJ981 | MG1655 carrying pBAD24*::*tm(*axe82*)-*eptA* | This work |
| FYJ982 | MG1655 carrying pBAD24*::*tm(*eptA*)-*axe82* | This work |
| FYJ983 | MG1655 carrying pBAD24*::*tm(*axe82*)-*mcr-1* | This work |
| FYJ984 | MG1655 carrying pBAD24*::*tm(*mcr-1*)-*axe82* | This work |
| FYJ915 | BL21 carrying pET21a*::mcr-1* | Lab stock |
| FYJ1097 | BL21(pLysS) carrying pET21a*::eptA* | Lab stock |
| FYJ1482 | BL21(pLysS) carrying pET21a*::axe82_07515* | This work |
| FYJ985 | BL21(pLysS) carrying pET21a*::*tm(*axe82*)-*eptA* | This work |
| FYJ986 | BL21(pLysS) carrying pET21a*::*tm(*eptA*)-*axe82* | This work |
| FYJ987 | BL21(pLysS) carrying pET21a*::*tm(*axe82*)-*mcr-1* | This work |
| FYJ988 | BL21(pLysS) carrying pET21a*::*tm(*mcr-1*)-*axe82* | This work |
| FYJ989 | BL21(pLysS) carrying pET21a*::axe82_07515* (E248A) | This work |
| FYJ990 | BL21(pLysS) carrying pET21a*::axe82_07515* (T287A) | This work |
| FYJ991 | BL21(pLysS) carrying pET21a*::axe82_07515* (H397A) | This work |
| FYJ992 | BL21(pLysS) carrying pET21a*::axe82_07515* (D472A) | This work |
| FYJ993 | BL21(pLysS) carrying pET21a*::axe82_07515* (H473A) | This work |
| FYJ994 | BL21(pLysS) carrying pET21a*::axe82_07515* (N110A) | This work |
| FYJ995 | BL21(pLysS) carrying pET21a*::axe82_07515* (T114A) | This work |
| FYJ996 | BL21(pLysS) carrying pET21a*::axe82_07515* (E118A) | This work |
| FYJ997 | BL21(pLysS) carrying pET21a*::axe82_07515* (S332A) | This work |
| FYJ998 | BL21(pLysS) carrying pET21a*::axe82_07515* (K335A) | This work |
| FYJ999 | BL21(pLysS) carrying pET21a*::axe82_07515* (H402A) | This work |
| FYJ1481 | BL21(pLysS) carrying pET21a*::axe82_07515* (H485A) | This work |
| **Plasmids** |  |  |
| pBAD24 | Arabinose inducible promoter-driven expression vector; Amp^R^ | Lab stock |
| pBAD24*::mcr-1* | A pBAD24 carrying the wild-type version of *mcr-1* at the two cuts of EcoRI and SalI; Amp^R^ | Lab stock |
| pBAD24*::eptA* | A pBAD24 carrying the wild-type version of *eptA* at the two cuts of EcoRI and SalI; Amp^R^ | Lab stock |
| pBAD24*::axe82_07515* | A pBAD24 carrying the wild-type version of *axe82_07515* at the two cuts of EcoRI and SalI; Amp^R^ | This work |
| pBAD24*::axe82_0751*5(E248A) | pBAD24 encoding the mutant version of  *axe82_0751*5 (E248A); Amp^R^ | This work |
| pBAD24*::axe82_0751*5(T287A) | pBAD24 encoding the mutant version of  *axe82_0751*5 (T287A); Amp^R^ | This work |
| pBAD24*::axe82_0751*5(H397A) | pBAD24 encoding the mutant version of  *axe82_0751*5 (H397A); Amp^R^ | This work |
| pBAD24*::axe82_0751*5(D472A) | pBAD24 encoding the mutant version of  *axe82_0751*5 (D472A); Amp^R^ | This work |
| pBAD24*::axe82_0751*5(H473A) | pBAD24 encoding the mutant version of  *axe82_0751*5 (H473A); Amp^R^ | This work |
| pBAD24*::axe82_0751*5(N110A) | pBAD24 encoding the mutant version of  *axe82_0751*5 (N110A); Amp^R^ | This work |
| pBAD24*::axe82_0751*5(T114A) | pBAD24 encoding the mutant version of  *axe82_0751*5 (T114A); Amp^R^ | This work |
| pBAD24*::axe82_0751*5(E118A) | pBAD24 encoding the mutant version of  *axe82_0751*5 (E118A); Amp^R^ | This work |
| pBAD24*::axe82_0751*5(S332A) | pBAD24 encoding the mutant version of  *axe82_0751*5 (S332A); Amp^R^ | This work |
| pBAD24*::axe82_0751*5(K335A) | pBAD24 encoding the mutant version of  *axe82_0751*5 (K335A); Amp^R^ | This work |
| pBAD24*::axe82_0751*5(H402A) | pBAD24 encoding the mutant version of  *axe82_0751*5 (H402A); Amp^R^ | This work |
| pBAD24*::axe82_0751*5(H485A) | pBAD24 encoding the mutant version of  *axe82_0751*5 (H485A); Amp^R^ | This work |
| pBAD24*::*tm(*axe82*)-  *eptA* | pBAD24 carrying a hybrid gene (i.e., the transmembrane region of *axe82_07515* fused with the extracellular domain of *eptA*); Amp^R^ | This work |
| pBAD24*::*tm(*eptA*)-  *axe82* | pBAD24 carrying a hybrid gene (i.e., the transmembrane region of *eptA* fused with the extracellular domain of *axe82_07515*); Amp^R^ | This work |
| pBAD24*::*tm(*axe82*)-  *mcr-1* | pBAD24 carrying a hybrid gene (i.e., the transmembrane region of *axe82_07515* fused with the extracellular domain of *mcr-1*); Amp^R^ | This work |
| pBAD24*::*tm(*mcr-1*)-  *axe82* | pBAD24 carrying a hybrid gene (i.e., the transmembrane region of *mcr-1* fused with the extracellular domain of *axe82_07515*; Amp^R^ | This work |
| pET21a | A T7 promoter-driven expression vector, Amp^R^ | Lab stock |
| pET21a*::eptA* | A pET21a carrying the wild-type version of *eptA* at the two cuts of NdeI and XhoI; Amp^R^ | Lab stock |
| pET21a*::mcr-1* | A pET21a carrying the wild-type version of *mcr-1* at the two cuts of NdeI and XhoI; Amp^R^ | Lab stock |
| pET21a*::axe82_07515* | A pET21a carrying the wild-type version of *axe82_07515* at the two cuts of NdeI and XhoI; Amp^R^ | This work |
| pET21a*::axe82_07515*  (E248A) | A pET21a carrying the wild-type version of *axe82_07515* (E248A) at the two cuts of NdeI and XhoI; AmpR | This work |
| pET21a*::axe82_07515*  (T287A) | A pET21a carrying the wild-type version of *axe82_07515* (T287A) at the two cuts of NdeI and XhoI; AmpR | This work |
| pET21a*::axe82_07515*  (H397A) | A pET21a carrying the wild-type version of *axe82_07515*(H397A) at the two cuts of NdeI and XhoI; AmpR | This work |
| pET21a*::axe82_07515*  (D472A) | A pET21a carrying the wild-type version of *axe82_07515*(D472A) at the two cuts of NdeI and XhoI; AmpR | This work |
| pET21a*::axe82_07515*  (H473A) | A pET21a carrying the wild-type version of *axe82_07515*(H473A) at the two cuts of NdeI and XhoI; AmpR | This work |
| pET21a*::axe82_07515*  (N110A) | A pET21a carrying the wild-type version of *axe82_07515*(N110A) at the two cuts of NdeI and XhoI; AmpR | This work |
| pET21a*::axe82_07515*  (T114A) | A pET21a carrying the wild-type version of *axe82_07515*(T114A) at the two cuts of NdeI and XhoI; AmpR | This work |
| pET21a*::axe82_07515*  (E118A) | A pET21a carrying the wild-type version of *axe82_07515* (E118A) at the two cuts of NdeI and XhoI; AmpR | This work |
| pET21a*::axe82_07515*  (S332A) | A pET21a carrying the wild-type version of *axe82_07515* (S332A) at the two cuts of NdeI and XhoI; AmpR | This work |
| pET21a*::axe82_07515*  (K335A) | A pET21a carrying the wild-type version of *axe82_07515* (K335A) at the two cuts of NdeI and XhoI; AmpR | This work |
| pET21a*::axe82_07515*  (H402A) | A pET21a carrying the wild-type version of *axe82_07515* (H402A) at the two cuts of NdeI and XhoI; AmpR | This work |
| pET21a*::axe82_07515*  (H485A) | A pET21a carrying the wild-type version of *axe82_07515* (H485A) at the two cuts of NdeI and XhoI; AmpR | This work |
| pET21a*::*tm(*axe82*)-  *eptA* | A pET21a carrying the transmembrane region of *axe82_07515* that is fused with the extracellular domain of *eptA* at the two cuts of NdeI and XhoI; Amp^R^ | This work |
| pET21a*::*tm(*eptA*)-  *axe82* | A pET21a carrying the transmembrane region of *eptA* that is fused with the extracellular domain of *axe82_07515* at the two cuts of NdeI and XhoI; Amp^R^ | This work |
| pET21a*::*tm(*axe82*)-  *mcr-1* | A pET21a carrying the transmembrane region of *axe82_07515* that is fused with the extracellular domain of *mcr-1* at the two cuts of NdeI and XhoI; Amp^R^ | This work |
| pET21a*::*tm(*mcr-1*)-  *axe82* | A pET21a carrying the transmembrane region of *mcr-1* fused to the extracellular domain of *axe82_07515* at the two cuts of NdeI and XhoI; Amp^R^ | This work |
