# Supplementary material for: Defining ICR-Mo, an intrinsic colistin resistance determinant from Moraxella osloensis
Source: PLoS Genet. 2018 May 14;14(5):e1007389. doi: 10.1371/journal.pgen.1007389 (PMC5983563; doi:10.1371/journal.pgen.1007389)
Supplement: S2 Table — (DOCX) [file pgen.1007389.s002.docx]

**Table S2** Primers used in this study

| **Primers** | **Sequences** |
| --- | --- |
| pBAD24-*axe82*-F(EcoRI) | 5’-AACC *GAATTC* ATG AGC GTG AAC AAC AGC CGT T-3’ |
| pBAD24-*axe82*-R(SalI) | 5’-CCG *GTCGAC* TTA CTT GAT AAA CAG CGG TTT ACC-3’ |
| pET21a-*axe82*-F  (NdeI) | 5’-GGAATTC *CATATG* AGC GTG AAC AAC AGC CGT T-3’ |
| pET21a-*axe82*-R  (XhoI) | 5’-CCG *CTCGAG* CTT GAT AAA CAG CGG TTT ACC-3’ |
| pBAD24-*eptA*-F  (EcoRI) | 5’-AACC *GAATTC* ATG ATA AAA CCG AAC CTG AGG CC-3’ |
| *eptA*-TM-R | 5’-CTC ACG AAA GAA GCT CGC ATA ATC CTG ATA TTG CAA CAT TG-3’ |
| *eptA*-OS-F | 5’-CGA TTC TGG CGA TGA GCA AAA ACT ACG CCT CGT TTT TCC GCA A-3’ |
| pBAD24-*eptA*-R  (SalI) | 5’-CCG *GTCGAC* TCA GCG CGG ACG GCG GCA GG-3’ |
| pET21a-*eptA*-F  (NdeI) | 5’-GGG AATTC *CATATG* ATA AAA CCG AAC CTG AGG CC-3’ |
| pET21a-*eptA*-R  (XhoI) | 5’-CCG *CTCGAG* GCG CGG ACG GCG GCA GGC T-3’ |
| *axe82*-TM-R | 5’-GTT TTT GCT CAT CGC CAG AAT CG-3’ |
| *axe82*-OS-F | 5’-TAT GCG AGC TTC TTT CGT GAG-3’ |
| *axe82*-N110A-F | 5’-TAA CAT GCT GCA GGC AGC GCT GCA AAC CGA TAA GG-3’ |
| *axe82*-N110A-R | 5’-CTG CCT GCA GCA TGT TAA CGT CAT ACA CGG TA-3’ |
| *axe82*-T114A-F | 5’-GCT GCA AGC AGA TAA GGC GGA AAG CGC GGA CC-3’ |
| *axe82*-T114A-R | 5’-CCT TAT CTG CTT GCA GCG CGT TCT GCA GCA TG-3’ |
| *axe82*-E118A-F | 5’-AAC CGA TAA GGC GGC AAG CGC GGA CCT GTT CAA CG-3’ |
| *axe82*-E118A-R | 5’-TTG CCG CCT TAT CGG TTT GCA GCG CGT TCT GC- 3’ |
| *axe82*-E248A-F | 5’-TTC GTG GTT GGT GCA ACC GCG CGT GGC GAC CAT-3’ |
| *axe82*-E248A-R | 5’-GTT GCA CCA ACC ACG AAA ACC ACC AGT TTC GG-3’ |
| *axe82*-T287A-F | 5’-TAC CAG CGC AGC GTA CAG CGT GCC GTG CAT GT-3’ |
| *axe82*-T287A-R | 5’-TGT ACG CTG CGC TGG TAC CGC ACG CAA TAA CC-3’ |
| *axe82*-S332A-F | 5’-CAA CAA CGC AAG CAG CAA GGG TGT TAC CAA CC-3’ |
| *axe82*-S332A-R | 5’-TGC TGC TTG CGT TGT TGT CAC GCC ACA GAA TG-3’ |
| *axe82*-K335A-F | 5’-AGC GCA GGT GTT ACC AAC CGT CTG CCG GCG GC-3’ |
| *axe82*-K335A-R | 5’-TTG GTA ACA CCT GCG CTG CTG CTG TTG TTG TCA CG-3’ |
| *axe82*-H397A-F | 5’-TCG TTC TGG CAC AGA TGG GTA ACC ACG GCC CG-3’ |
| *axe82*-H397A-R | 5’-CAT CTG TGC CAG AAC GAT CAG GGT ATC TTG GT-3’ |
| *axe82*-H402A-F | 5’-ACC AGA TGG GTA ACG CAG GCC CGG CGT ACT TTA AGC-3’ |
| *axe82*-H402A-R | 5’-TGC GTT ACC CAT CTG GTG CAG AAC GAT CAG GG-3’ |
| *axe82*-D472A-F | 5’-TAT GTT AGC GCA CAC GGC GAG AGC CTG GGT GA-3’ |
| *axe82*-D472A-R | 5’-CCG TGT GCG CTA ACA TAC AGC ATC GCC ACC TG-3’ |
| *axe82*-H473A-F | 5’-TAT GTT AGC GAC GCA GGC GAG AGC CTG GGT GAA-3’ |
| *axe82*-H473A-R | 5’-CCT GCG TCG CTA ACA TAC AGC ATC GCC ACC TG-3’ |
| *axe82*-H485A-F | 5’-ATC TAC CTG GCA GGC ATG CCG TAT AAA ATT GCG-3’ |
| *axe82*-H485A-R | 5’-ATG CCT GCC AGG TAG ATG CCG TTT TCA CCC AG-3’ |

*The underlined letters in italic denote restrictions sites
