# Supplementary material for: Defining ICR-Mo, an intrinsic colistin resistance determinant from Moraxella osloensis
Source: PLoS Genet. 2018 May 14;14(5):e1007389. doi: 10.1371/journal.pgen.1007389 (PMC5983563; doi:10.1371/journal.pgen.1007389)
Supplement: S3 Table — (DOC) [file pgen.1007389.s003.doc]

**Table S3** Comparative analyses of secondary structure motifs among the EptA, MCR-1, ICR-Mo and their derivatives

| Protein* | Percentage of secondary structure motifs (%) | | | |
| --- | --- | --- | --- | --- |
|  | α-helix | β-sheet | η-turn | Coils |
| EptA (PDB: 5FGN) | 50.7 | 11.8 | 18.7 | 18.8 |
| EptA | 55.8 | 5.0 | 16.7 | 22.5 |
| MCR-1 | 55.6 | 5.2 | 16.6 | 22.6 |
| ICR-Mo | 57.4 | 3.7 | 17.3 | 21.6 |
| TM(AXE82)-EptA | 57.0 | 4.4 | 20.6 | 18.0 |
| TM(EptA)-AXE82 | 55.3 | 6.0 | 17.3 | 21.4 |
| TM(AXE82)-MCR-1 | 54.7 | 5.3 | 16.7 | 23.3 |
| TM(MCR-1)-AXE82 | 47.6 | 12.2 | 16.8 | 23.4 |

*The detailed information on secondary structure motifs in EptA is highlighted in blue, which is calculated according to its resolved crystal structure of full-length protein (PDB: 5FGN). All the other data are estimated through circular dichroism-based measurement of the proteins.
