# Supplementary figures and images for: Defining ICR-Mo, an intrinsic colistin resistance determinant from Moraxella osloensis
Source: PLoS Genet. 2018 May 14;14(5):e1007389. doi: 10.1371/journal.pgen.1007389 (PMC5983563; doi:10.1371/journal.pgen.1007389)

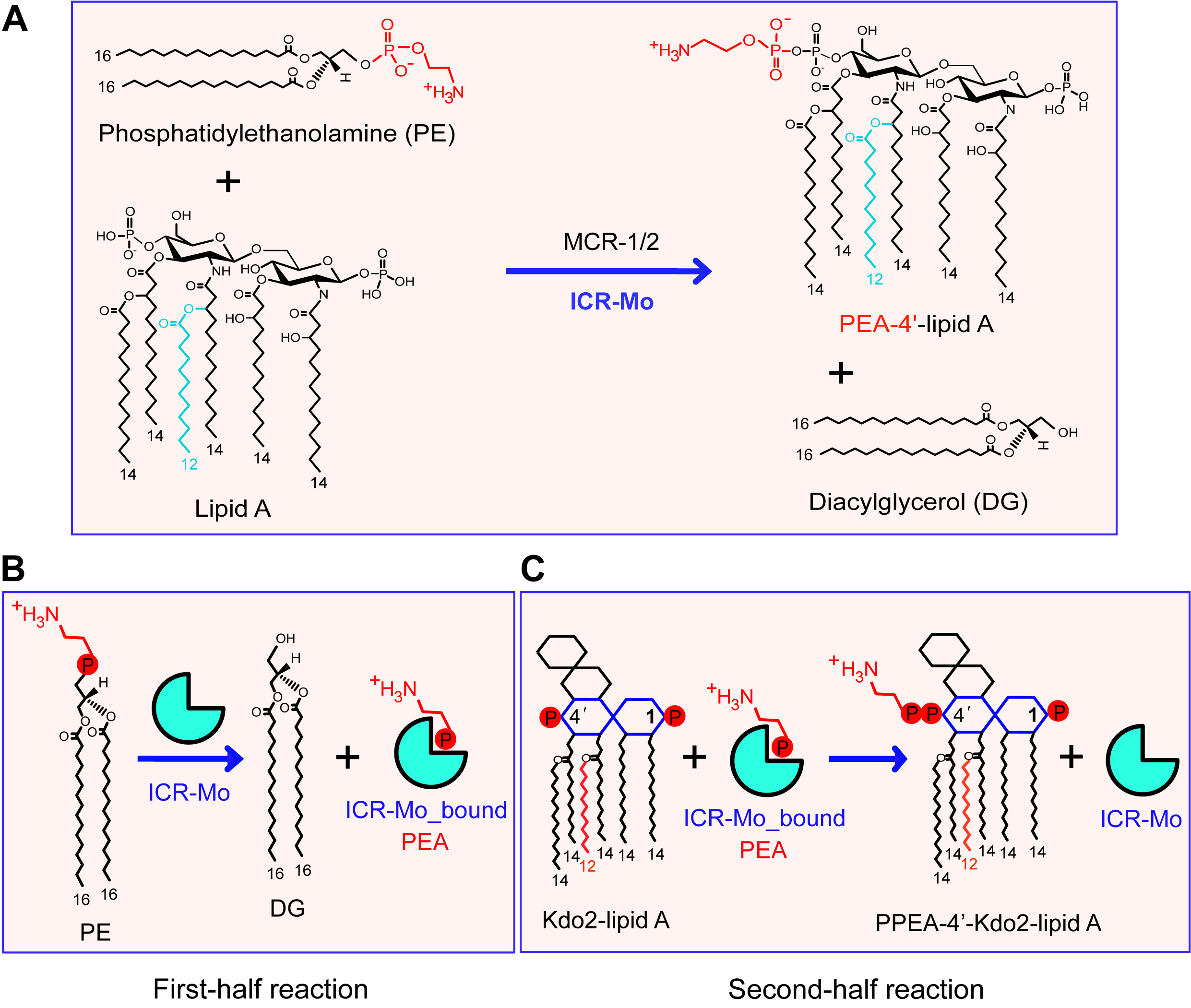

Supplement: S1 Fig — A. Chemical reaction proposed for the transfer of PEA to lipid A by ICR-Mo. ICR-Mo catalyzes the addition of PEA to the 1 or 4’ -position of lipid A moiety anchored on lipopolysaccharides, in which final products referred to PEA-4’-lipid A and diacylglycerol. The chemical structures of molecules are depicted with ChemDraw software. B. First-half reaction proposed for the ICR-Mo catalysis is illustrated with the removal of PEA from PE, giving the final product DG and an adduct of ICR-Mo-bound PEA. C. Second-half reaction proposed for ICR-Mo catalysis, which involves the generation of the final product Kdo2-lipid A-1 (or 4’)-PPEA through the transfer of the intermediate product ICR-Mo-bound PEA to the recipient Kdo2-lipid A. (TIF) [file pgen.1007389.s004.tif]

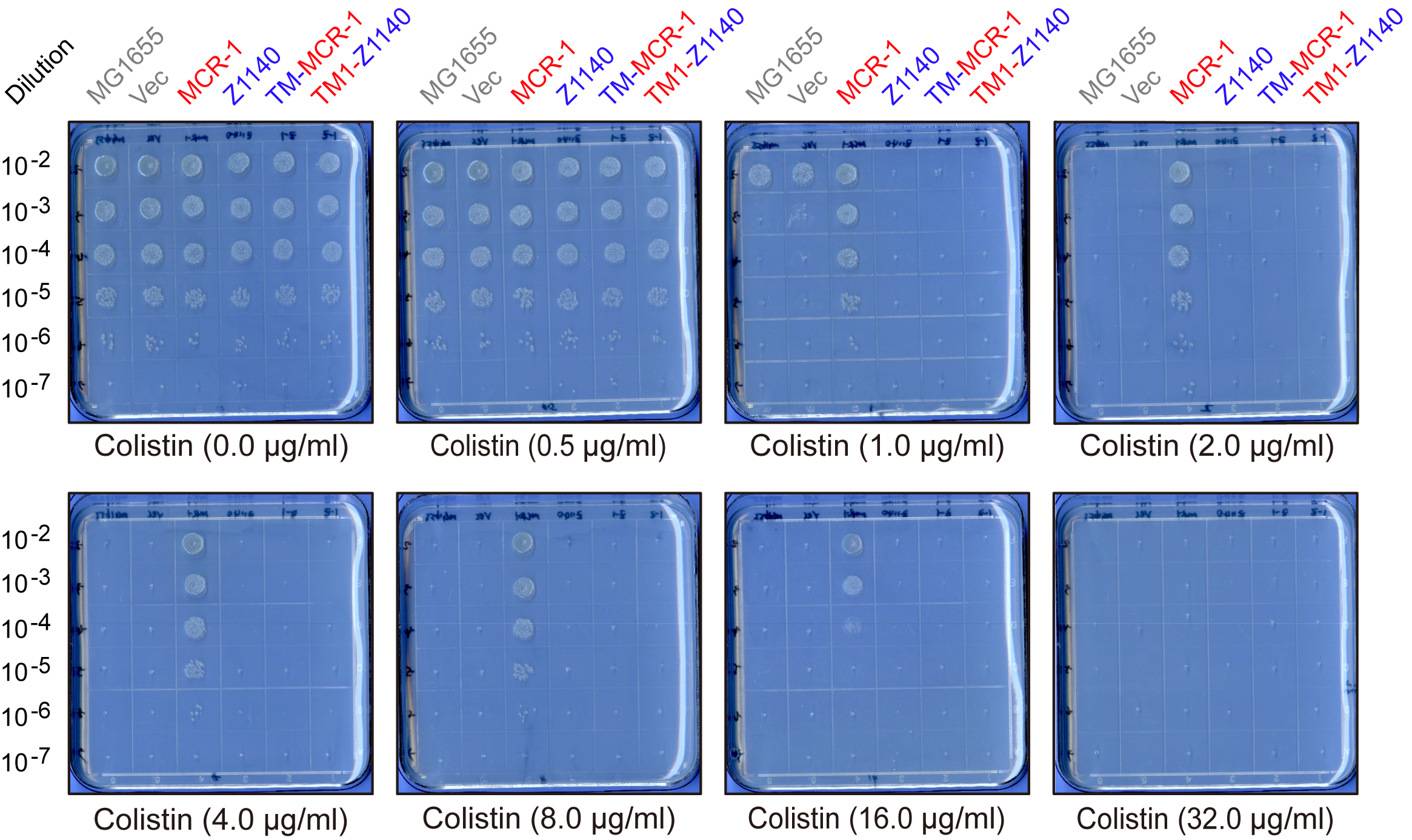

Supplement: S2 Fig — The E. coli MG1655 cultures in mid-log phase were spotted on LBA plates with different levels of colistin (0, 0.5, 1.0, 2.0, 4.0, 8.0, 16.0, and 32.0 μg/ml) and maintained for 20 hrs at 37°C. Prior to bacterial spotting, 0.2% arabinose was mixed into the melted media of LBA to trigger expression of pBAD24-based MCR-like genes as we described [86, 87]. Designations: Vec, the arabinose-inducible pBAD24 vector; TM-MCR-1, a hybrid version of MCR-1 in which the TM region is replaced with the counterpart in Z1140 of the E. coli O157:H7 strain EDL933; TM1-Z1140, a mosaic derivative of Z1140 carrying the TM domain of MCR-1.Here, it seems likely that Z1140 and its two domain-swapped versions (TM-MCR-1 & TM1-Z1140) have no roles in conferring the colistin-susceptible MG1655 resistance to polymyxin. A representative photograph of three independent assays is given. (TIF) [file pgen.1007389.s005.tif]

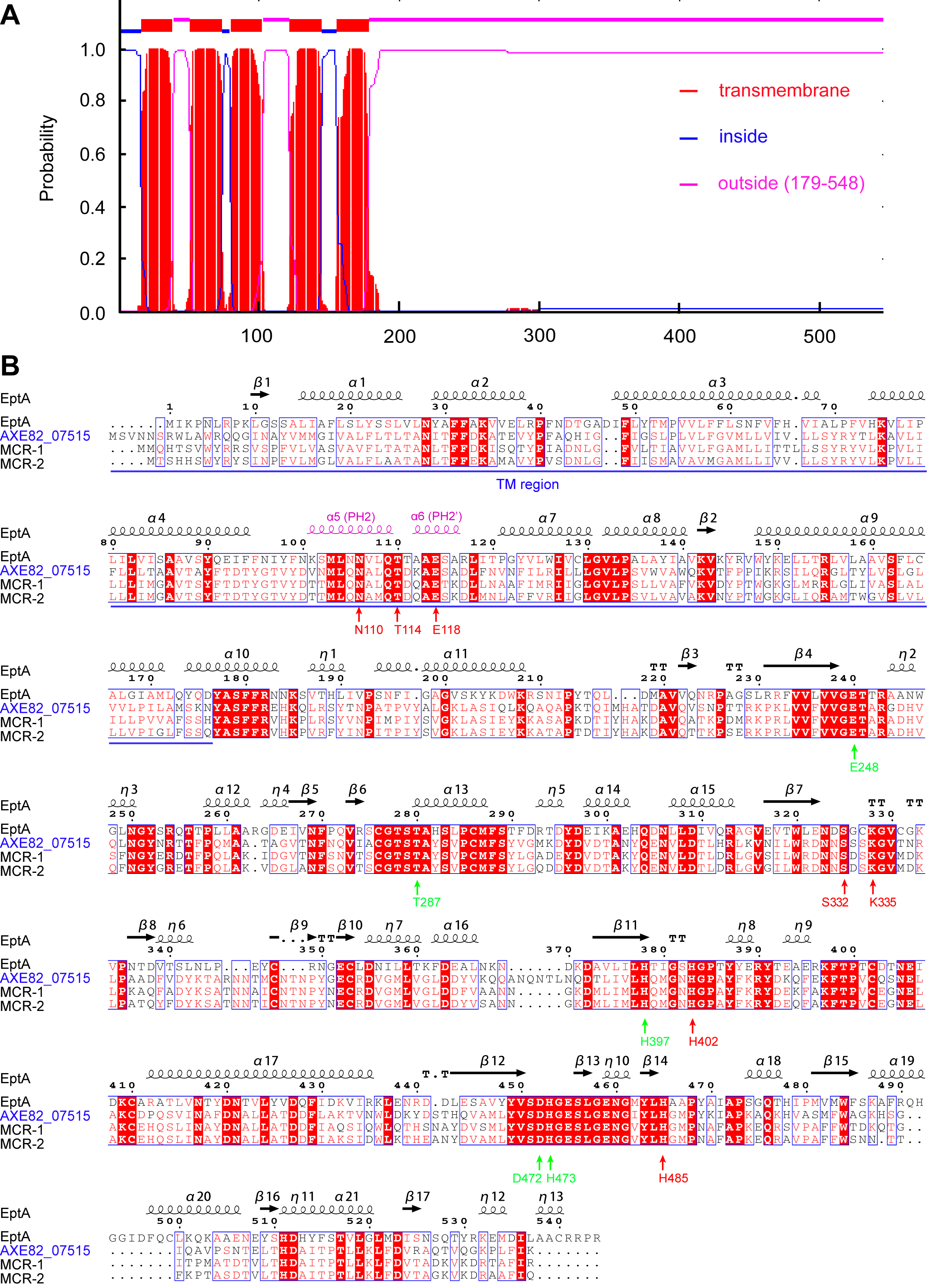

Supplement: S3 Fig — A. Transmembrane prediction of ICR-Mo. B. Sequence comparison of ICR-Mo with EptA and MCR-1/2 homologs. The polypeptide sequences of four enzymes (EptA, ICR-Mo, MCR-1 and MCR-2) were subjected to Clustal Omega (http://www.ebi.ac.uk/Tools/msa/clustalo/) for sequence alignments. The final output is generated with the program ESPript 3.0 (http://espript.ibcp.fr/ESPript/cgi-bin/ESPript.cgi) [22]. The trans-membrane (TM) region was underlined in blue following the prediction with TMHMM server v2.0 (http://www.cbs.dtu.dk/services/TMHMM) and underlined in blue. The five Zn2+-interacting residues (E248, T287, H397, D472 and H473) are indicated with green arrows, and the seven PE substrate-binding residues highlighted with red arrows included N110, T114, E118, S332, K335, H402, and H485, respectively. Identical residues are in white letters with red background, similar residues are in red letters with white background, and the varied residues are in black letters. The protein secondary structure was shown in cartoon (on top). Designations: α: α-helix; β: β-sheet; T: Turn; η: coil; PH: Periplasmic-facing helix. (TIF) [file pgen.1007389.s006.tif]

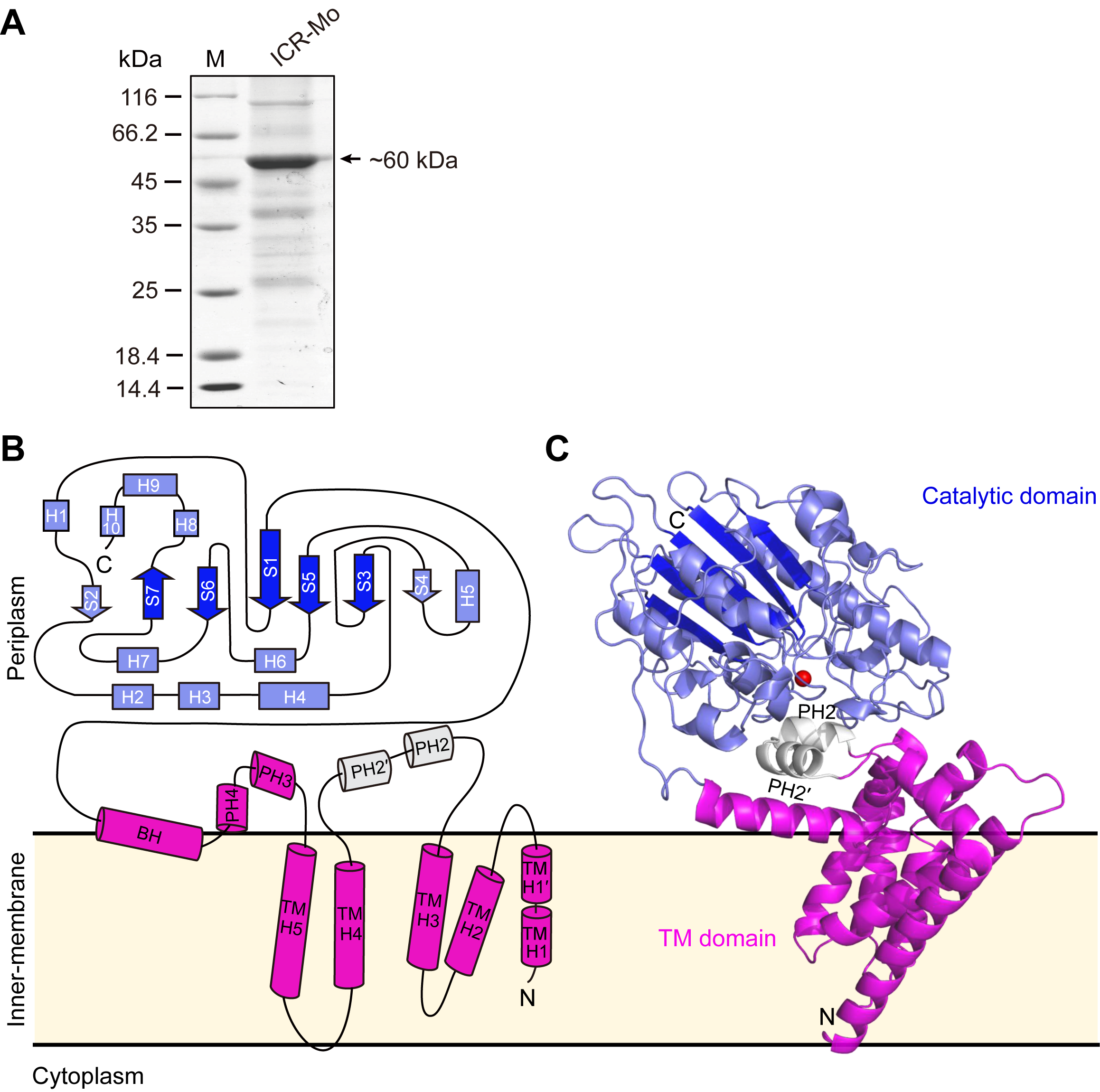

Supplement: S4 Fig — A. SDS-PAGE (12%) profile of the purified ICR-Mo protein. B. Scheme for the ICR-Mo topology. C. Overall architecture of the full-length ICR-Mo integral membrane protein. The overall structure of ICR-Mo (AXE82_07515) in ribbon was modeled using the Neisseria meningitis EptA (PDB: 5FGN) as structural template. The catalytic domain is illustrated in blue, the TM region is highlighted in magenta and the two helices (PH2 and PH2’) are in light grey. The rectangle with light peach background refers to the layer of inner-membrane. The red sphere denotes zinc ion. Designations: TM, Trans-membrane; PH, Periplasmic-facing helices; H, α-helix; S, β-sheet; N, N-terminus; C, C-terminus. (TIF) [file pgen.1007389.s007.tif]

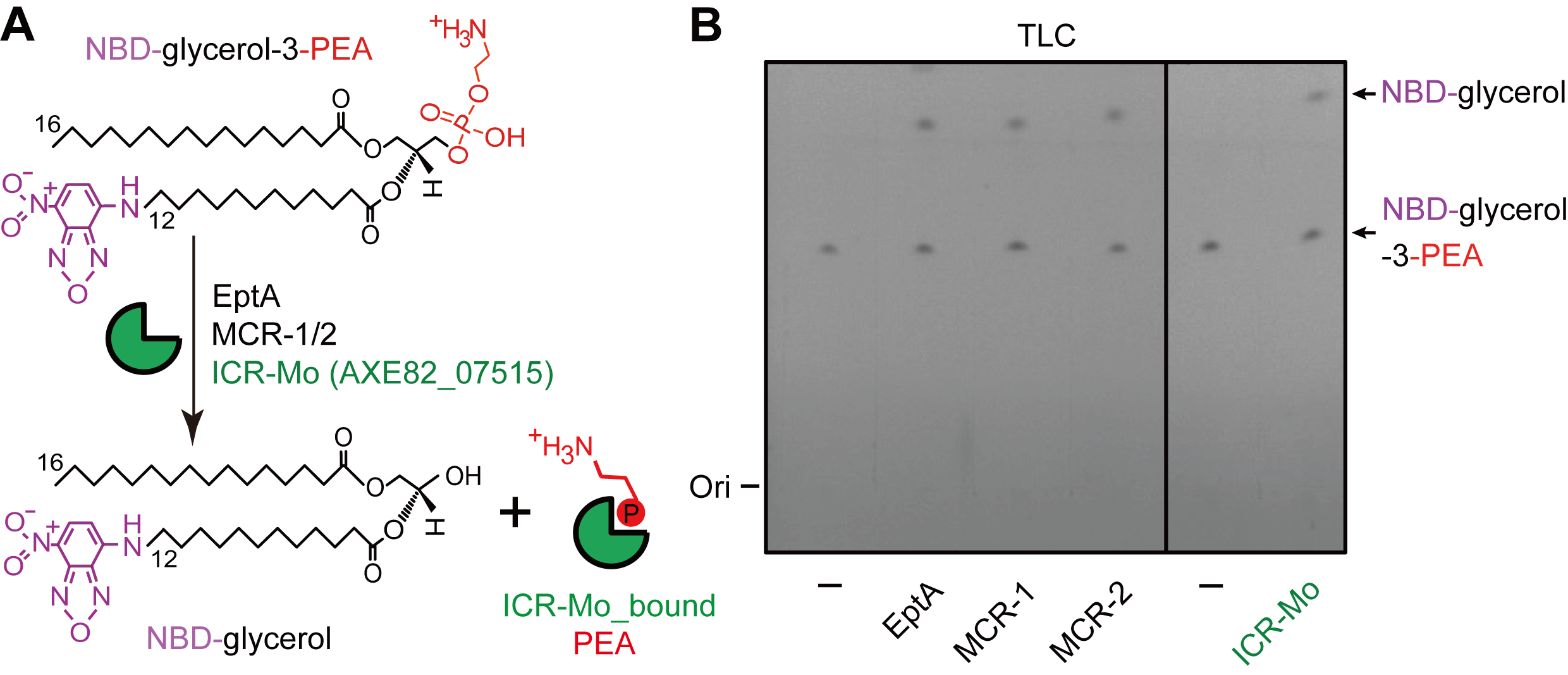

Supplement: S5 Fig — A. Scheme for ICR-Mo-based hydrolysis reaction of NBD-glycerol-3-PEA into NBD-glycerol. B. TLC analyses of the alternative substrate NBD-glycerol-3-PEA and its resultant product NBD-glycerol. Thin layer chromatography (TLC) was performed as Anandan et al. [34] described with minor changes. The identities of both NBD-glycerol-3-PEA and NBD-glycerol were validated with LC/MS (seen in Fig 1). Minus denotes no addition of the enzyme of EptA/MCR-1/MCR-2/ICR-Mo. (TIF) [file pgen.1007389.s008.tif]

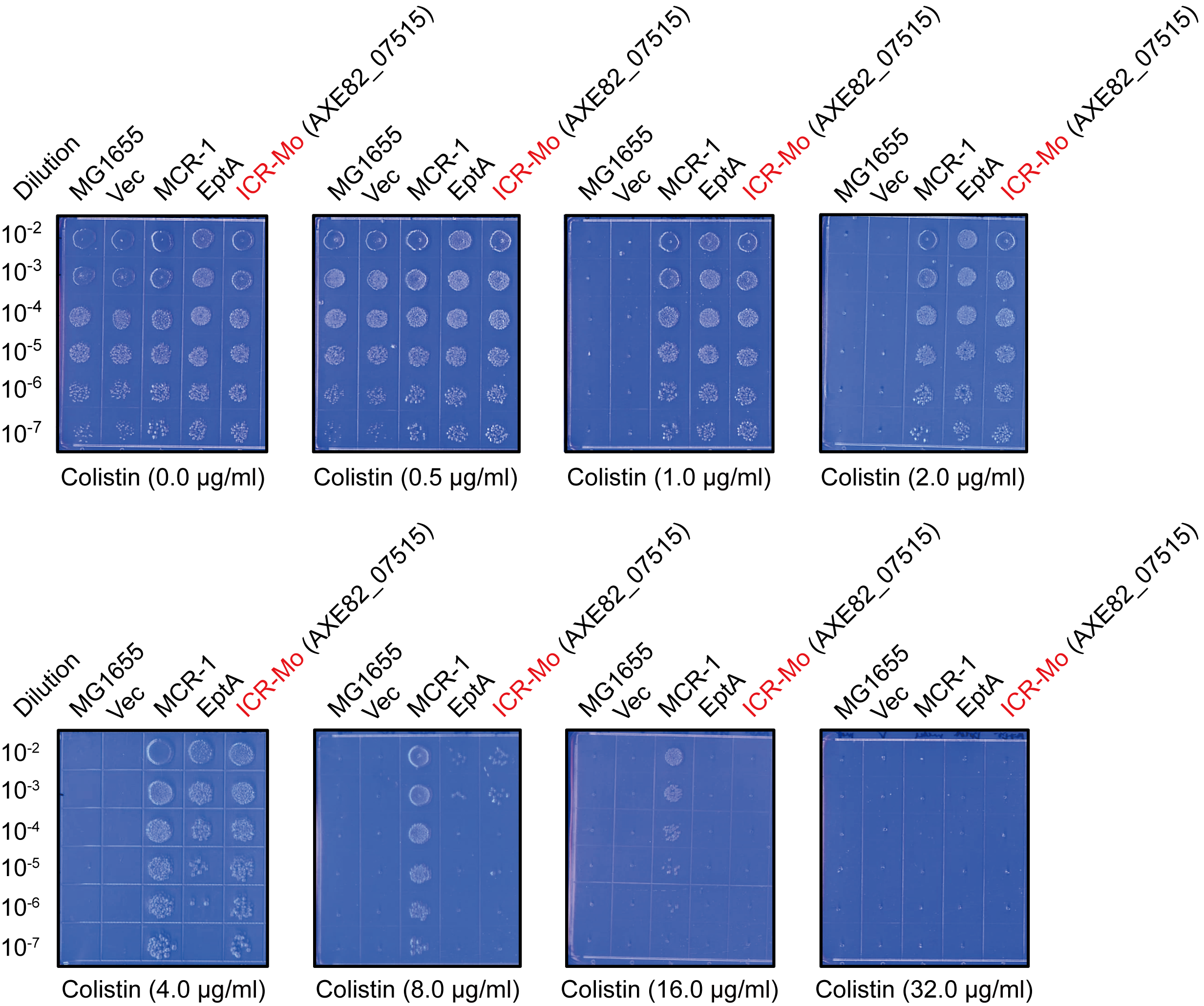

Supplement: S6 Fig — The serial diluted cultures (in mid-log phase) were spotted on LBA plates with various concentrations of colistin (0, 0.5, 1.0, 2.0, 4.0, 8.0, 16.0, and 32.0 μg/ml) and maintained at 37°C for ~20 hrs. Plasmid-borne expression of icr-Mo (axe82_07515)/eptA/mcr-1 is dependent on the arabinose-inducible expression vector pBAD24 in E. coli MG1655. 0.2% arabinose is supplied here as an inducer. A representative result of three independent tests is given. (TIF) [file pgen.1007389.s009.tif]

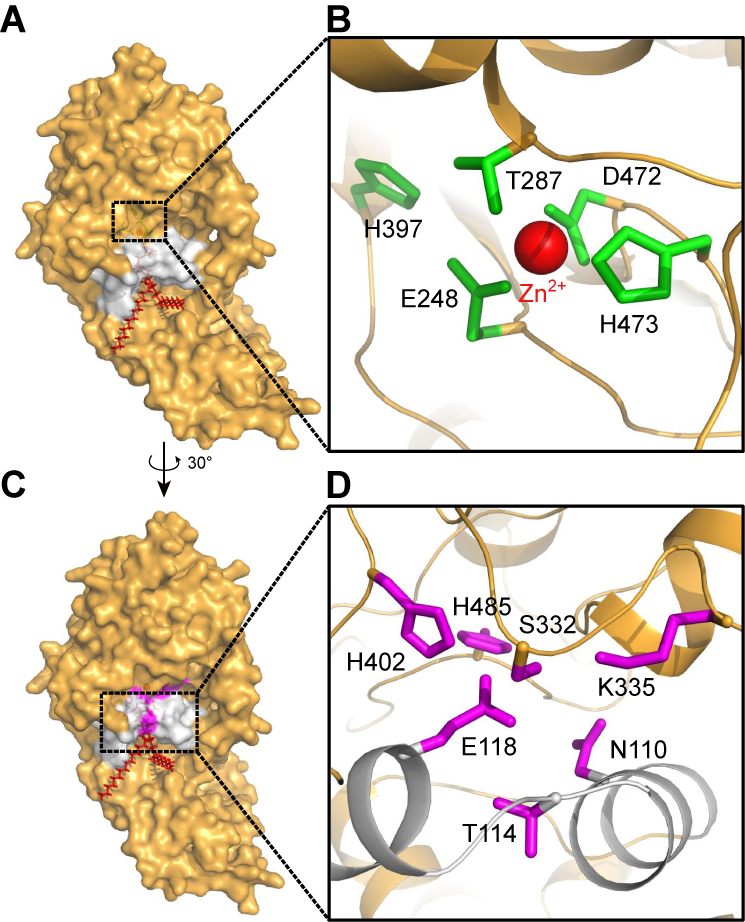

Supplement: S7 Fig — A. Surface structure of ICR-Mo with the cavity required for entry and binding of PE substrate. B. An enlarged illustration for a five residues-containing, Zn2+-binding motif. The five residues in Zn2+-binding motif of ICR-Mo refers to E248, T287, H397, D472 and H473, respectively. C. Surface structure of ICR-Mo in counter-clockwise rotation (30°). D. Fine structural illustration of the seven residues-containing motif that is involved in binding of ICR-Mo to PE lipid substrate. The seven residues corresponded to N110, T114, E118, S332, K335, H402 and H485, respectively. (TIF) [file pgen.1007389.s010.tif]

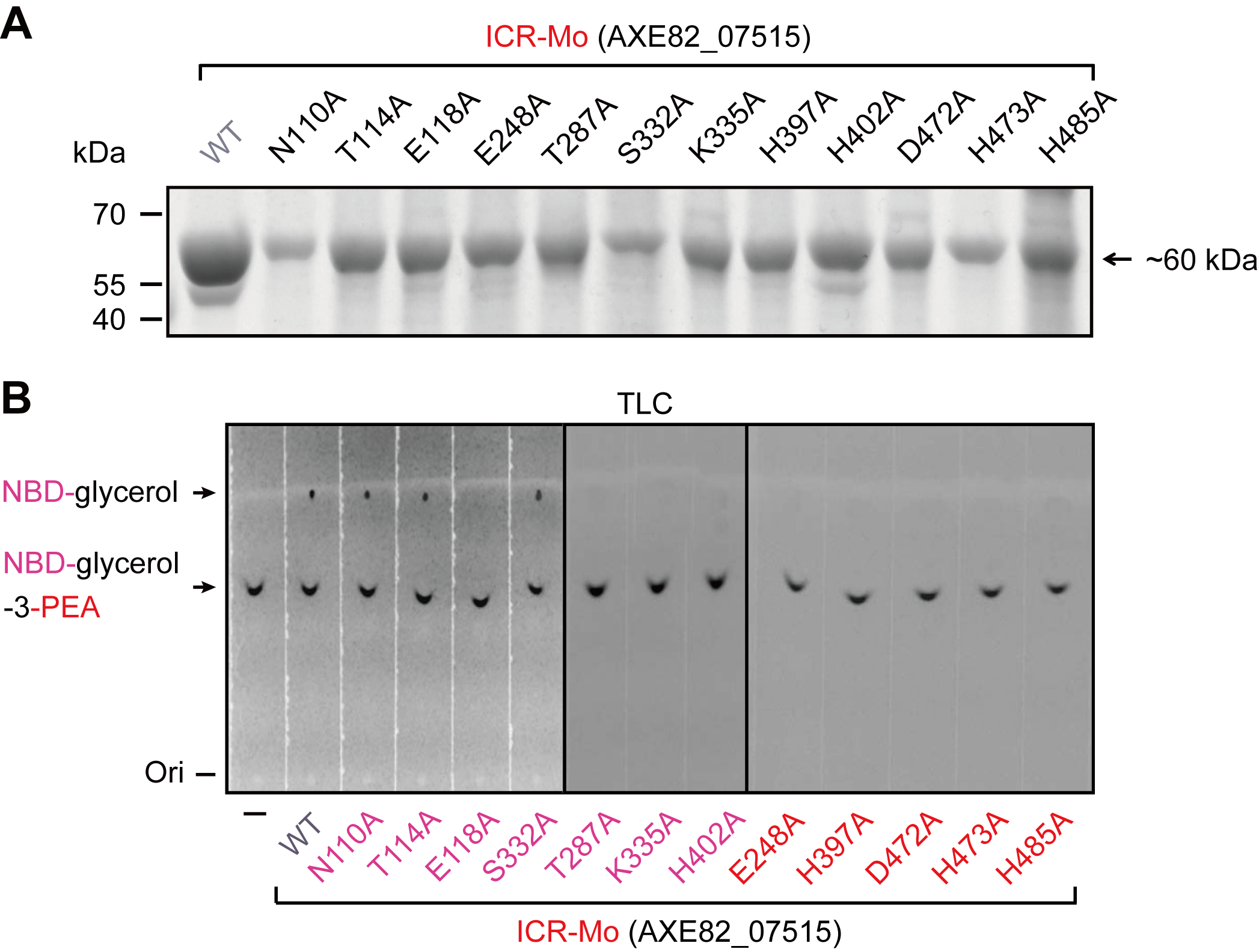

Supplement: S8 Fig — A. SDS-PAGE (12%) profile of the purified ICR-Mo transmembrane enzyme and its 12 derivatives. The estimated mass of ICR-Mo (and its derivatives) is ~60 kDa, indicated with an arrow. B. TLC-based analyses of in vitro enzymatic activities of ICR-Mo and its 12 point-mutants. The identities of both NBD-glycerol-3-PEA and NBD-glycerol were validated by LC/MS (seen in Fig 1). This suggests that only three point-mutants of ICR-Mo (N110A, T114A & S332A) retain partial enzymatic activities to hydrolyze the alternative substrate NBD-glycerol-3-PEA into NBD-glycerol. TLC experiments were conducted as described by Anandan et al. [34] with minor modifications. A representative result from three independent trials is given, and this photograph was generated through combining three different TLC plates with limited wells. (TIF) [file pgen.1007389.s011.tif]

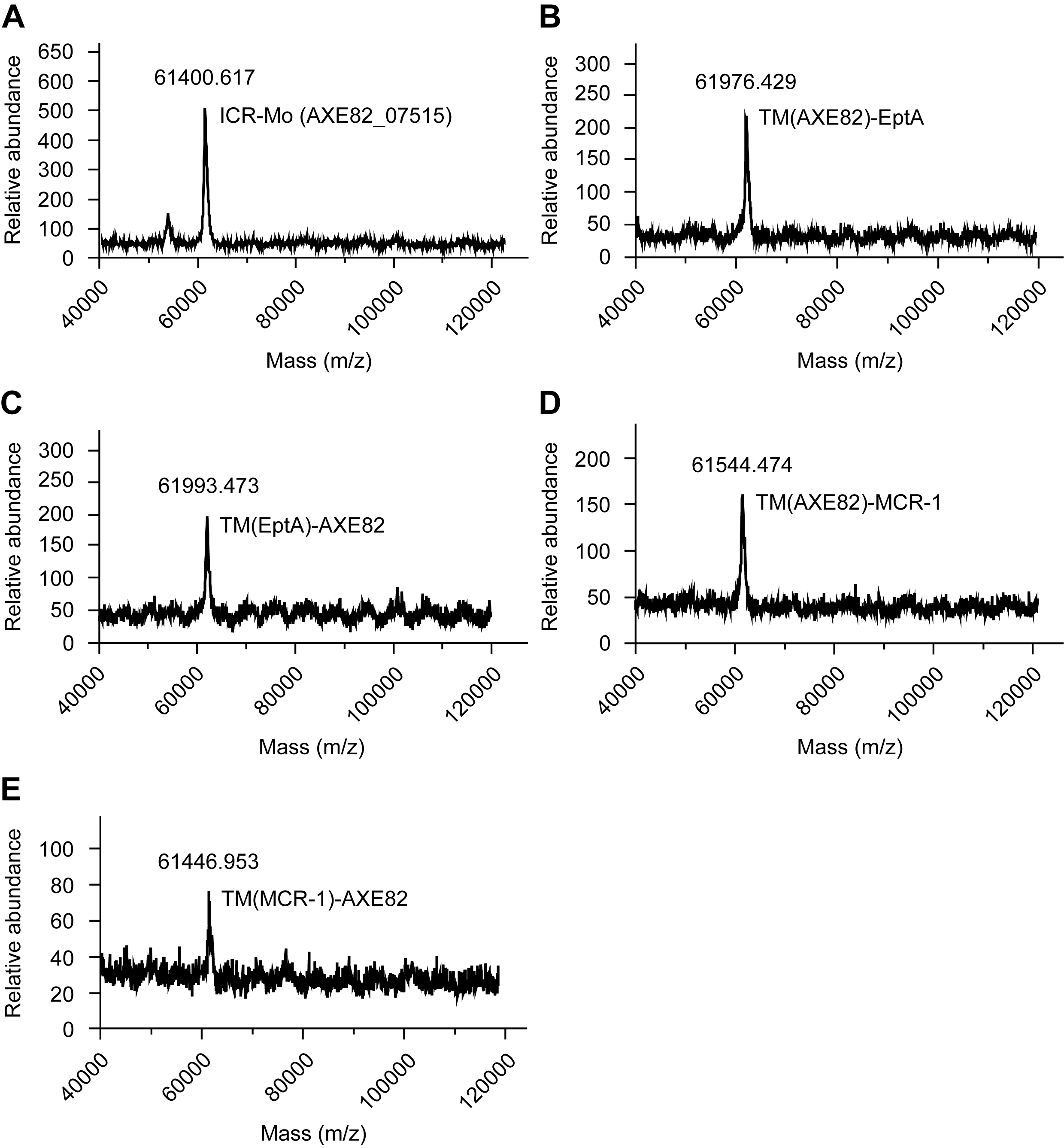

Supplement: S9 Fig — A. MALDI-TOF determination of molecular weight of ICR-Mo protein. B. Use of MALDI-TOF to measure molecular mass of an intact hybrid version of ICR-Mo protein, TM(AXE82)-EptA. C. Molecular mass of TM(EptA)-AXE82 (a mosaic derivative of AXE82_07515 protein) revealed by MALDI-TOF. D. Molecular mass of a hybrid version of ICR-Mo (AXE82_07515) protein, TM(AXE82)-MCR-1. E. Molecular weight of TM(MCR-1)-AXE82, a hybrid version of AXE82_07515 (TIF) [file pgen.1007389.s012.tif]

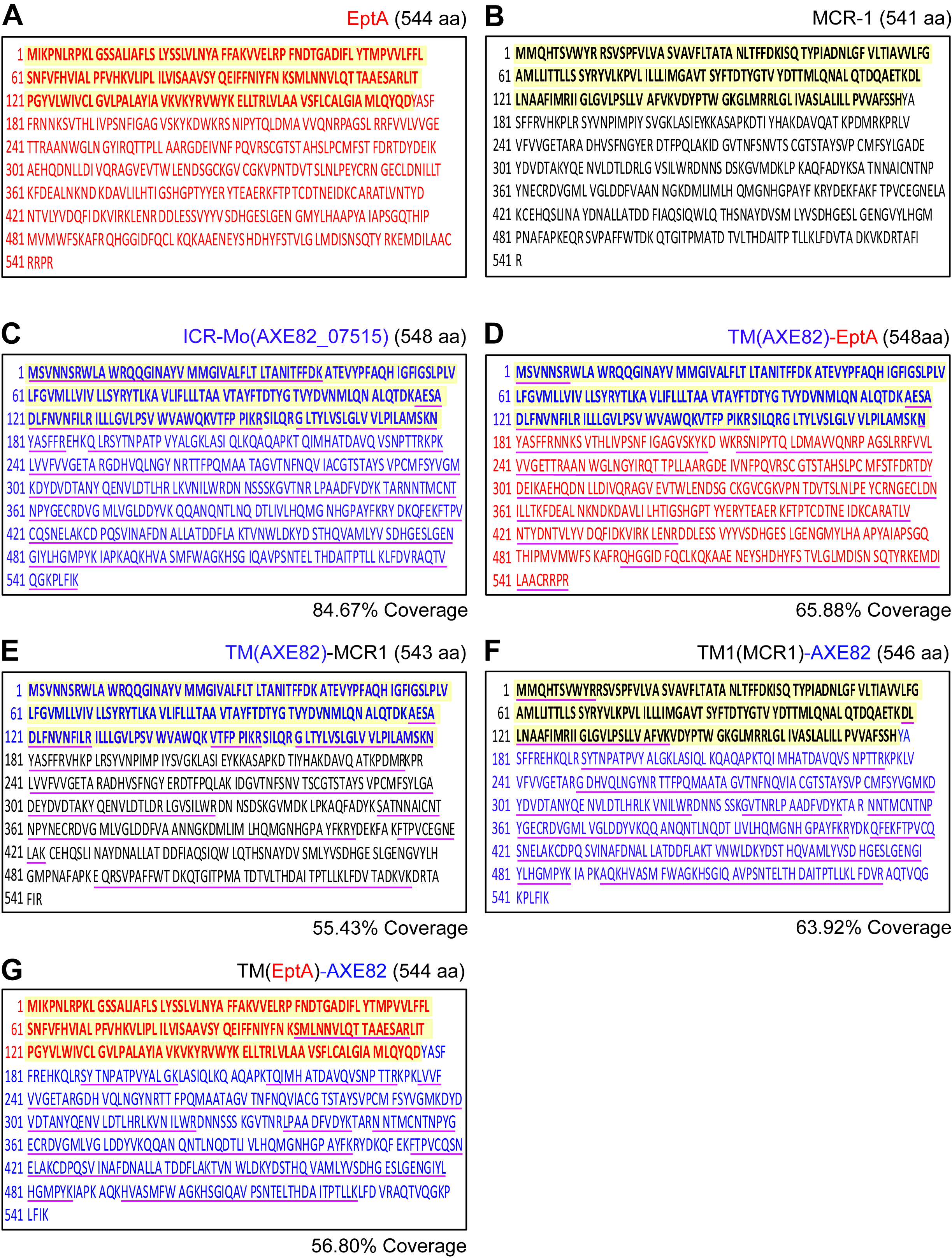

Supplement: S10 Fig — A. Protein sequence of Neisseria gonorrhea EptA. B. Protein sequence of MCR-1. C. MS-based identification of ICR-Mo. D. MS verification of the chimeric version of ICR-Mo, TM(AXE82)-EptA. E. MS determination of a hybrid version of ICR-Mo, TM(AXE82)-MCR-1. F. MS-based elucidation of the mosaic version of ICR-Mo, TM1(MCR-1)-AXE82. G. MS identification of the hybrid deriivative of ICR-Mo, TM(EptA)-AXE82. The bold letters with yellow background denote the TM regions, and the other letters refer to catalytic domains. The underlined letters correspond to the polypeptides identified by mass spectrometry. Designations: TM(AXE82)-EptA, a derivative of AXE82_07515 whose extracellular region is replaced with its counterpart in EptA; TM(AXE82)-MCR-1, a derivative of AXE82_07515 whose extracellular region is replaced with its counterpart in MCR-1; TM1(MCR-1)-AXE82, a derivative of AXE82_07515 whose TM region is replaced with its counterpart in MCR-1; TM(EptA)-AXE82, a derivative of AXE82_07515 in which TM region is replaced with that of EptA. (TIF) [file pgen.1007389.s013.tif]

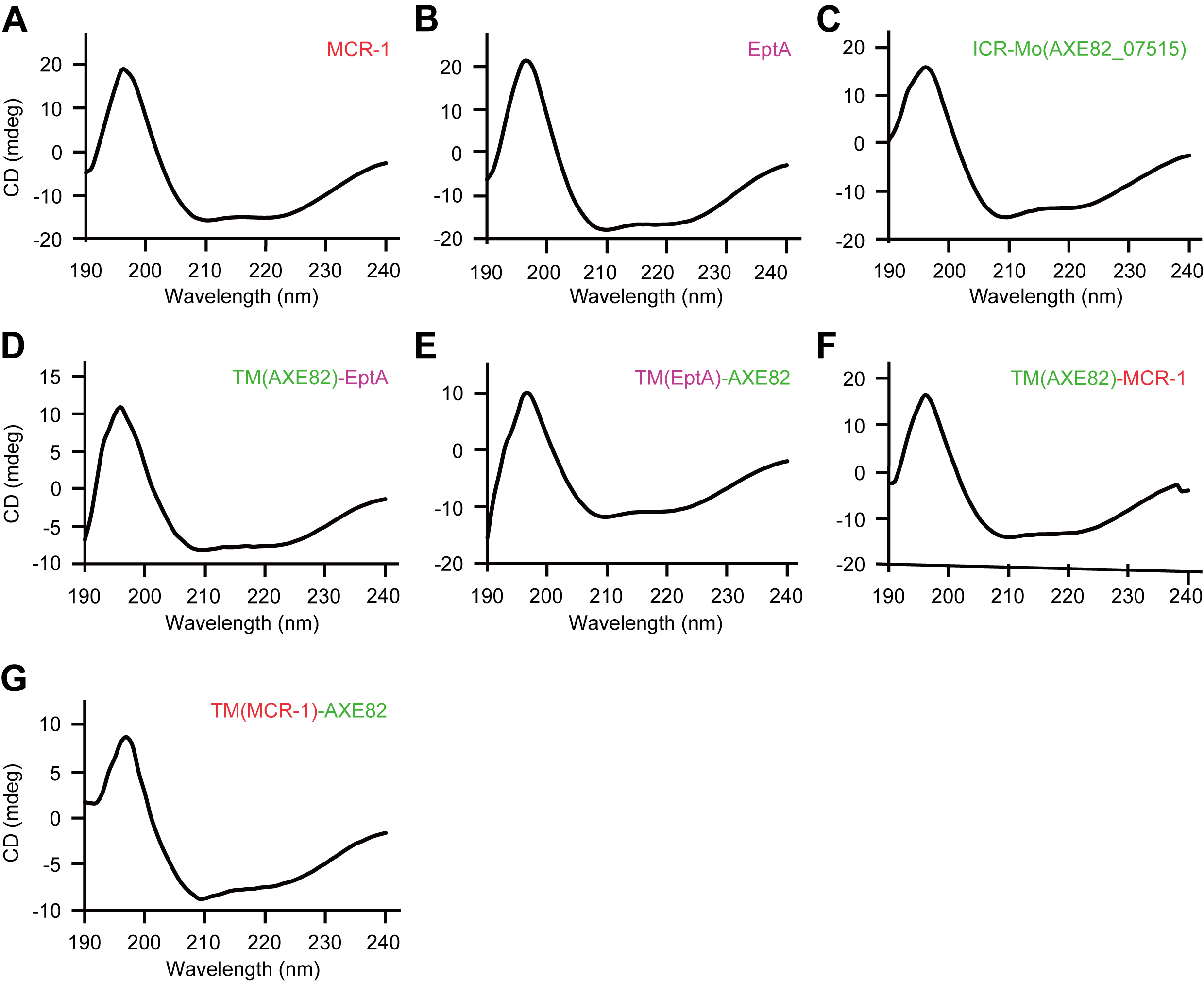

Supplement: S11 Fig — A. CD-based visualization of secondary structure of MCR-1 protein. B. CD spectrum of EptA protein. C. CD assays for ICR-Mo. D. CD spectrum of the ICR-Mo derivative, TM(AXE82)-EptA. E. CD spectrum of the hybrid version of AXE82_07515, TM(EptA)-AXE82. F. CD spectrum of the mosaic version of AXE82_07515, TM(AXE82)-MCR-1. G. CD profile of the mosaic version of AXE82_07515, TM(MCR-1)-AXE82. Here, the CD results of EptA/MCR-1/ICR-Mo and their domain-swapped versions indicate they share a similar conformation in protein secondary structures. Abbreviations: CD, Circular dichroism; TM(AXE82)-EptA, a derivative of AXE82_07515 whose extracellular region is replaced with its counterpart in EptA (Panel B); TM(EptA)-AXE82, a derivative of AXE82_07515 whose TM region is replaced with its counterpart in EptA (Panel B); M(AXE82)-MCR-1, a derivative of AXE82_07515 whose extracellular region is replaced with its counterpart in MCR-1 (Panel A); TM1(MCR-1)-AXE82, a derivative of AXE82_07515 whose TM region is replaced with its counterpart in MCR-1 (Panel A). (TIF) [file pgen.1007389.s014.tif]

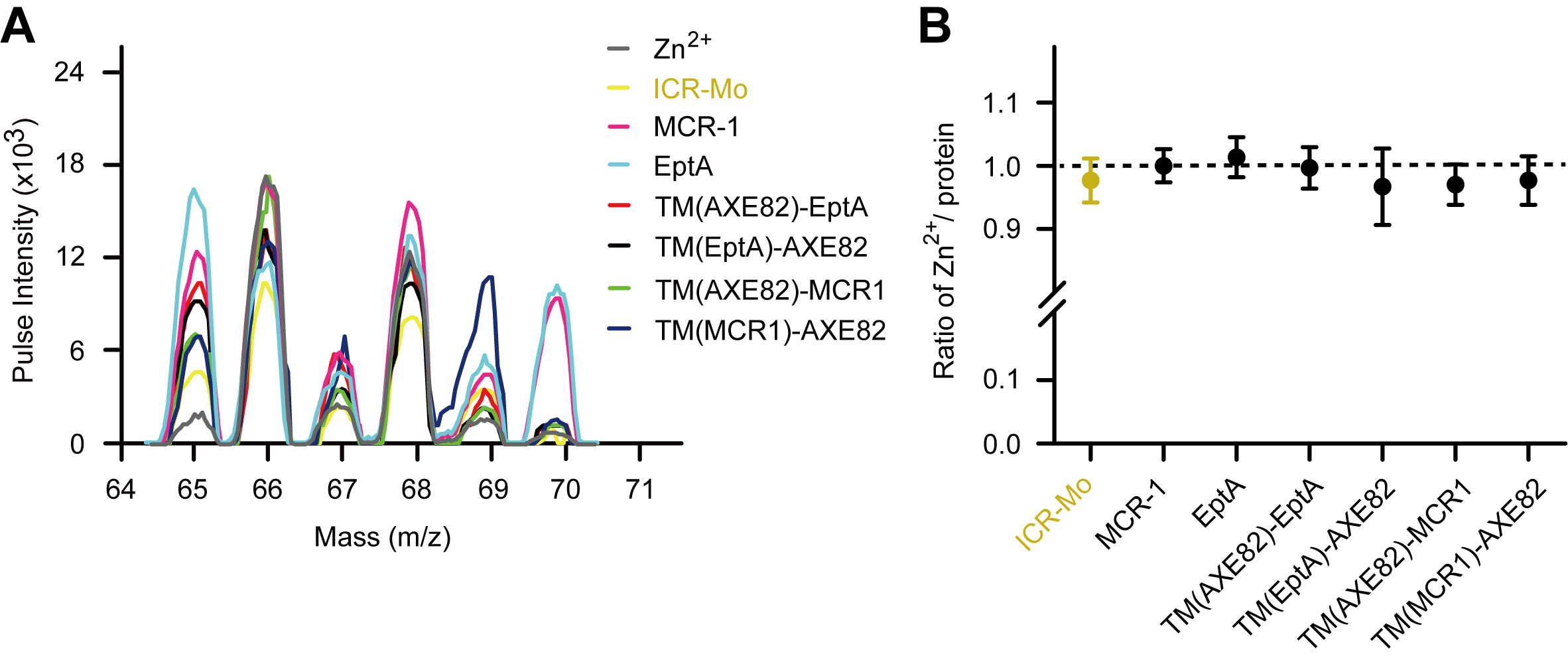

Supplement: S12 Fig — A. Inductively coupled plasma mass spectrometry (ICP/MS)-based detection of Zn2+ in ICR-Mo/MCR-1/EptA and the related chimeric derivatives. B. Use of ICP/MS to calculate the relative ratio of protein-bound Zn2+ to protein. Similar to the observations we recently reported [36, 37], ICP/MS data of ICR-Mo confirms that it might possess zinc ions at the relative ratio of 1:1 (zinc: protein). Indeed, it is also consistent with scenarios seen with the crystal structure of the neissertial EptA [20, 34]. The designations of ICR-Mo derivatives are identical to those of S11 Fig. (TIF) [file pgen.1007389.s015.tif]
